# Overcoming *EGFR*^G724S^-mediated osimertinib resistance through unique binding characteristics of second-generation EGFR inhibitors

**DOI:** 10.1038/s41467-018-07078-0

**Published:** 2018-11-07

**Authors:** Jana Fassunke, Fabienne Müller, Marina Keul, Sebastian Michels, Marcel A. Dammert, Anna Schmitt, Dennis Plenker, Jonas Lategahn, Carina Heydt, Johannes Brägelmann, Hannah L. Tumbrink, Yannic Alber, Sebastian Klein, Alena Heimsoeth, Ilona Dahmen, Rieke N. Fischer, Matthias Scheffler, Michaela A. Ihle, Vanessa Priesner, Andreas H. Scheel, Svenja Wagener, Anna Kron, Konrad Frank, Katia Garbert, Thorsten Persigehl, Michael Püsken, Stefan Haneder, Bernhard Schaaf, Ernst Rodermann, Walburga Engel-Riedel, Enriqueta Felip, Egbert F. Smit, Sabine Merkelbach-Bruse, H. Christian Reinhardt, Stefan M. Kast, Jürgen Wolf, Daniel Rauh, Reinhard Büttner, Martin L. Sos

**Affiliations:** 1 Institute of Pathology, University Hospital of Cologne, Kerpener Str. 62, 50937 Cologne, Germany; 2 Molecular Pathology, Institute of Pathology, University Hospital of Cologne, Kerpener Str. 62, 50937 Cologne, Germany; 3 Department of Translational Genomics, Center of Integrated Oncology Cologne–Bonn, Medical Faculty, University of Cologne, Weyertal 115b, 50931 Cologne, Germany; 4 Faculty of Chemistry and Chemical Biology, TU Dortmund University, Otto-Hahn-Str. 4a, 44227 Dortmund, Germany; 5 Lung Cancer Group Cologne and Network Genomic Medicine (Lung Cancer), Department I of Internal Medicine, Center for Integrated Oncology Cologne-Bonn, University Hospital Cologne, Kerpener Str. 62, 50931 Cologne, Germany; 6 Center for Molecular Medicine Cologne, University of Cologne, Robert-Koch-Str. 21, 50931 Cologne, Germany; 7 Department I of Internal Medicine, University Hospital of Cologne, Weyertal 115b, 50931 Cologne, Germany; 8 Cologne Excellence Cluster on Cellular Stress Response in Aging-Associated Diseases, University of Cologne, Joseph Stelzmann Str. 26, 50931 Cologne, Germany; 9 Else Kröner Forschungskolleg Clonal Evolution in Cancer, University Hospital Cologne, Weyertal 115b, 50931 Cologne, Germany; 10 Section Pneumology, Clinic III of Internal Medicine, University Hospital of Cologne, Kerpener Str. 62, 50937 Cologne, Germany; 11 Institute of Diagnostic and Interventional Radiology, University Hospital of Cologne, Kerpener Str. 62, 50937 Cologne, Germany; 12 Hospital Dortmund gGmbH, Muensterstrasse 240, 44145 Dortmund, Germany; 13 Onkologie Rhein-Sieg, Schloßstraße 18, 53840 Troisdorf, Germany; 14 Department of Pneumology, Lung Hospital Cologne Merheim, City of Cologne Municipal Hospitals, Cologne, Germany; 15 Oncology Department, Vall d’Hebron University Hospital, Barcelona, Spain; 16 Thoracic Oncology Service, Netherlands Cancer Institute, Plesmanlaan 121, 1066 CX Amsterdam, The Netherlands

## Abstract

The emergence of acquired resistance against targeted drugs remains a major clinical challenge in lung adenocarcinoma patients. In a subgroup of these patients we identified an association between selection of *EGFR*^T790M^-negative but *EGFR*^G724S^-positive subclones and osimertinib resistance. We demonstrate that *EGFR*^G724S^ limits the activity of third-generation EGFR inhibitors both in vitro and in vivo. Structural analyses and computational modeling indicate that *EGFR*^G724S^ mutations may induce a conformation of the glycine-rich loop, which is incompatible with the binding of third-generation TKIs. Systematic inhibitor screening and in-depth kinetic profiling validate these findings and show that second-generation EGFR inhibitors retain kinase affinity and overcome *EGFR*^G724S^-mediated resistance. In the case of afatinib this profile translates into a robust reduction of colony formation and tumor growth of *EGFR*^G724S^-driven cells. Our data provide a mechanistic basis for the osimertinib-induced selection of *EGFR*^G724S^-mutant clones and a rationale to treat these patients with clinically approved second-generation EGFR inhibitors.

## Abstract (web summary)

Acquired resistance to targeted drugs remains a major clinical challenge in lung adenocarcinoma patients. Here, the authors show how the acquired *EGFR*^G724S^ mutation induces resistance to third-generation EGFR inhibitors and why the mutant kinase remains susceptible to second-generation inhibitors.

## Introduction

The identification of *EGFR* mutations and the discovery of their exquisite sensitivity to epidermal growth factor receptor (EGFR) inhibitors dramatically changed the therapeutic routine for lung adenocarcinoma (LADC) patients^1–3^. Selective inhibition of EGFR with tyrosine kinase inhibitors (TKI), such as erlotinib or gefitinib, significantly prolongs the progression-free survival (PFS) up to 13.6 months in the first-line setting^4–6^. However, under therapeutic pressure resistant clones emerge in virtually all tumors and ultimately lead to progressive disease and failure of therapy^7–9^.

Third-generation EGFR inhibitors such as osimertinib have been designed to overcome acquired resistance induced by the *EGFR*^T790M^ gatekeeper mutation^10^. Clinical results show that patients treated with osimertinib respond in up to 71% in the background of an acquired *EGFR*^T790M^ mutation^11,12^. More recent data indicate that osimertinib treatment is even superior to single agent first-generation inhibitors such as erlotinib or gefitinib in terms of PFS and overall survival (OS) in the first-line setting^13^.

The recurrent acquisition of *EGFR*^C797S^ mutations is currently thought to be the most frequent mechanism of resistance to osimertinib^14–16^. Alternative by-pass mechanisms involving *MET* amplification or activation of the MAPK pathway may also play a role in the development of resistance to third-generation EGFR inhibitors^14,15,17^. Here, we characterized the role of the acquired *EGFR*^G724S^ mutation that was diagnosed in osimertinib-resistant lesions of four individual *EGFR*^19del^-mutant LADC patients. We performed systematic biochemical, cellular, and structural analyses to determine the functional relevance of this mutation in the context of targeted EGFR inhibition.

## Results

### Acquisition of *EGFR*^G724S^ is associated with cancer progression

**Fig. 1:**
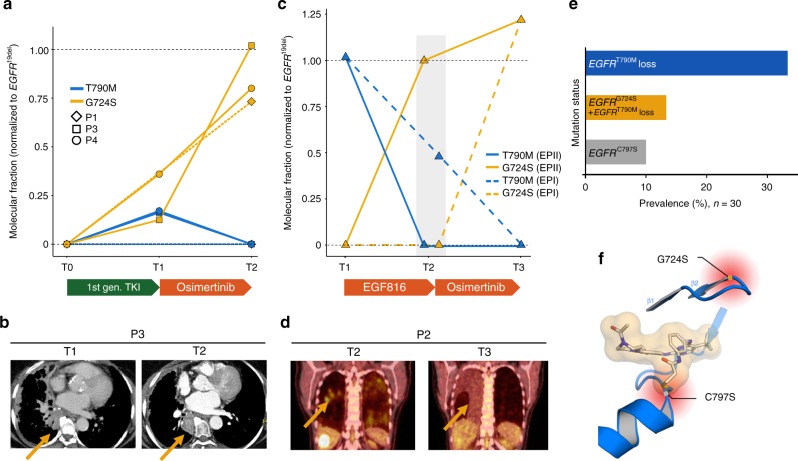
Insight into patient’s examination results and prevalence of *EGFR* resistance mutations. **a** Graph showing the molecular fractions of *EGFR*^T790M^ and *EGFR*^G724S^ normalized to *EGFR*^19del^ at first diagnosis (T0), prior to treatment with osimertinib (T1) and at progression to osimertinib (T2) in patients P1, P3, and P4. Treatment is indicated with bars below. All patients received a first-generation EGFR inhibitor (1st gen. TKI) prior to treatment with osimertinib. In P1, next-generation sequencing was not feasible at T1 and *EGFR* status was determined by Sanger sequencing (dotted lines). **b** The contrast-enhanced CT scans of patient P3 prior to treatment with osimertinib (T1) and at progression (T2) are exemplarily shown. The yellow arrows mark the spot of the biopsy collected in a growing lesion. **c** Prior to the start of osimertinib treatment (T2) two separate biopsies were collected (EPI, dotted line and EPII, solid line). Graph indicates the evolution of the molecular fractions of *EGFR*^T790M^ and *EGFR*^G724S^ in patient P2 prior to treatment with EGF816 (T1) and osimertinib (T2) and at progression to osimertinib (T3). **d** ^18^FDG PET-CT scans shows P2 prior to treatment (T2) and at progression to treatment with osimertinib (T3). **e** Bar chart showing the frequencies of *EGFR* mutations (*EGF*R^G724S^ and *EGFR*^C797S^) at progression to third-generation EGFR inhibition (*n* = 30). **f** Positions of the osimertinib resistance mutations *EGFR*^C797S^ and *EGFR*^G724S^ within the binding site of the EGFR kinase domain are shown (PDB ID: 5UWD)

Within our LADC re-biopsy program we performed targeted sequencing of lesions that progressed under treatment with third-generation EGFR inhibitors. Interestingly, we identified two patients with no detectable *EGFR*^G724S^ reads (P1, *EGFR*^E746_S752delinsV^; P2, *EGFR*^S752_I759del^) and two patients with low levels of *EGFR*^G724S^ mutation (P3, *EGFR*^E746_T751delinsIP^; P4, *EGFR*^E746_T751delinsIP^) prior to start of third-generation EGFR inhibitor therapy (Fig. 1a–d; Supplementary Fig. 1A, Supplementary Table 1). Patient P1 (UICC stage IIIA, 59 years old, female) received osimertinib within the AURA trial (NCT01802632) after progression on erlotinib and the detection of an acquired *EGFR*^T790M^ mutation (T1) (Fig. 1a). Osimertinib treatment resulted in a partial response (54% reduction based on RECIST 1.1) (Supplementary Fig. 1B, 1D). Even though progression occurred after 8.2 months with the growth of target lesions and a new *EGFR*^T790M^-negative and *EGFR*^G724S^-positive pleural effusion with a molecular fraction (MF, estimate of allelic fraction without calculating the purity and ploidy) of 6.3% (T2) (Supplementary Table 1C).

Patient P2 (UICC stage IV, 47 years old, female) received two consecutive lines of third-generation EGFR inhibitors upon progression to erlotinib and a combination of carboplatin/pemetrexed/bevacizumab. Treatment with the third-generation EGFR inhibitor EGF816 (CEGF816X2101; NCT02108964) was initiated after detection of *EGFR*^T790M^ (Fig. 1c). EGF816 treatment resulted in a stable disease according to RECIST 1.1 for almost 6 months. At the time point of progression to EGF816 two pulmonal lesion were resected (T2). In one (EPII), the initially detected *EGFR*^T790M^ mutation was lost and an *EGFR*^G724S^ mutation was acquired with a MF of 71.1%. In the other sample (EPI), the *EGFR*^T790M^ mutation (AF 39.3%) was still present and no *EGFR*^G724S^ reads were detected (Fig. 1c, Supplementary Table 1). Osimertinib treatment was initiated and resulted in a metabolic response of the liver metastases and the stabilization of other solid tumor lesions, including the remaining pulmonary lesions, as assessed by ^18^FDG positron emission tomography (PET)-CT scans (Fig. 1d). However, a progressive malignant pleural effusion that contained *EGFR*^G724S^-mutant cells and no trace of the previously acquired *EGFR*^T790M^ mutation was recorded after seven months of treatment (T3). In this example the MF of *EGFR*^G724S^ exceeds the MF of our reference *EGFR*^19del^ reads but it is likely that the inherent noise of our PCR-based method overestimates the *EGFR*^G724S^ reads and we assume similar levels of both mutants in this tumor^18^.

Two patients (P3, *EGFR*^E746_T751delinsIP^; P4, *EGFR*^E746_T751delinsIP^) were identified with a low MF of *EGFR*^G724S^ before initiation of osimertinib treatment and persisting *EGFR*^G724S^-mutant reads at time of progression (Fig. 1a, Supplementary Table 1). Patient P3 (68 years old, UICC stage IV, female) showed a robust response to gefitinib and at time of progression after 32 months presented with a growing paravertebral *EGFR*^T790M^-positive lesion (MF of 6.9%, T1) as well as an *EGFR*^G724S^ mutation (MF of 5.3%, T1; Supplementary Table 1). Treatment with osimertinib resulted in a good partial response according to RECIST 1.1 for a period of 6.8 months (Supplementary Fig. 1C). However, the lesion in which the *EGFR*^G724S^ mutation was detected showed no significant decrease. A subsequent re-biopsy of the same paravertebral lesion showed a loss of *EGFR*^T790M^ and the persistence of *EGFR*^G724S^ (MF 49.6%, T2) (Fig. 1b).

The last patient (P4) (69 years old, UICC stage IV) with a known mutation was initially treated with erlotinib (Fig. 1a, Supplementary Fig. 1A, Supplementary Table 1). However, progression occurred after 36 months of treatment and a re-biopsy of a growing lesion revealed *EGFR*^T790M^- as well as *EGFR*^G724S^-positive sequencing reads (T1). Treatment with osimertinib resulted in a PFS of 2.5 months (objective efficacy not determined by RECIST 1.1). Another re-biopsy of the growing lesion in the left lower lobe was collected revealing the loss of *EGFR*^T790M^ and the outgrowth of a *EGFR*^G724S^-mutant subclone (AF 39.3%, T2) (Supplementary Table 1). Thus, although *EGFR*^G724S^-positive clones may be partially selected in tumors treated with first-generation EGFR inhibitors, a pronounced increase of *EGFR*^G724S^-positive sequencing reads is primarily associated with third-generation EGFR inhibitor treatment.

*EGFR*^G724S^ mutations have been identified as very rare driver mutations and more recently, case reports have shown their potential role in acquired osimertinib resistance in LADC patients^19–21^. To assess the overall frequency of the acquired *EGFR*^G724S^ mutation and other changes in EGFR, we revisited re-biopsy samples obtained from *EGFR*^T790M^-positive patients at time of progression under treatment with third-generation EGFR inhibitors. This cohort spans 30 patients; 22 of them received osimertinib (73.3%), four EGF816 (13.3%) and four rociletinib (13.3%). *EGFR*^C797S^ was detected in three patients (10%), loss of *EGFR*^T790M^ without no detectable *EGFR* acquired mutation was detected in 10 (33.3%) and loss of *EGFR*^T790M^ and presence of *EGFR*^G724S^ was seen in four patients (13.3%) (Fig. 1e). As this mutation seems to be less frequent in other cohorts, the actual prevalence across a broader panel of patients with acquired osimertinib resistance remains to be assessed^14^. Although only *EGFR*^C797S^ mutations have a direct impact on the binding of third-generation EGFR inhibitors within the kinase, the mutual exclusivity between *EGFR*^C797S^ and *EGFR*^G724S^ indicates that the *EGFR*^G724S^ mutations do not represent passenger events (Fig. 1f).

Overall, our data show that *EGFR*^G724S^ mutations may emerge or persist in osimertinib-resistant clones that may evolve independently of acquired *EGFR*^T790M^ mutations. The data further suggest a negative relation of the allelic frequencies of *EGFR*^G724S^ and *EGFR*^T790M^ under third-generation EGFR inhibition: increasing *EGFR*^G724S^ frequencies were accompanied by decreasing *EGFR*^T790M^ frequencies.

### EGFR^G724S^ mediates resistance to third-generation EGFR inhibitors

**Fig. 2:**
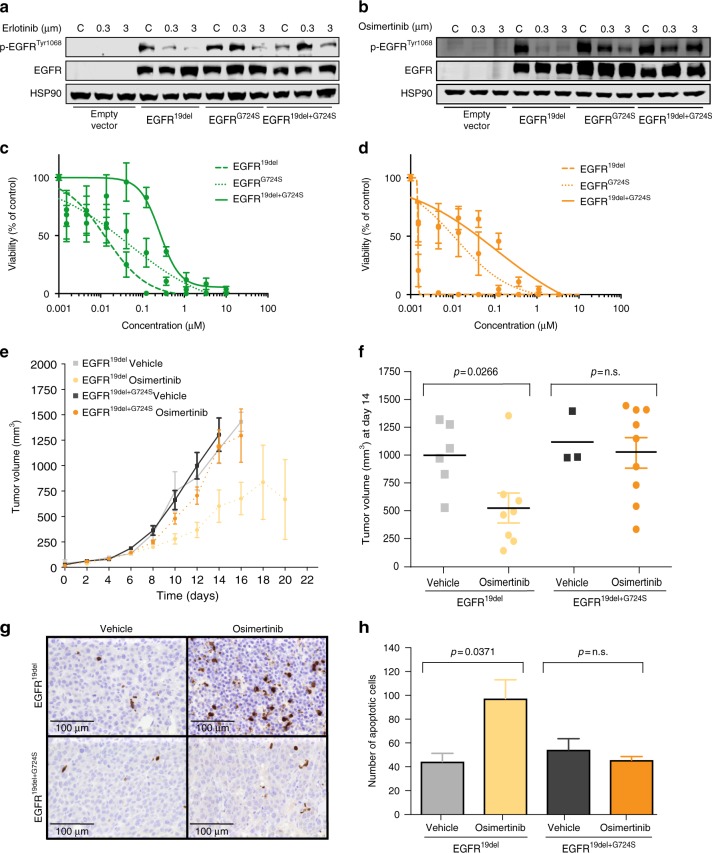
*EGFR*^G724S^ mediates resistance to third-generation EGFR inhibitors in vitro and in vivo. **a** Immunoblotting results of NIH-3T3 cells (empty vector, *EGFR*^19del^, *EGFR*^G724S^, or *EGFR*^19del+G724S^) monitoring phospho-EGFR and total EGFR under erlotinib treatment (24 h). HSP90 was used as loading control. **b** Immunoblotting of NIH-3T3 cells under osimertinib treatment (24 h) is shown. Dose–response measurement of Ba/F3 cells expressing *EGFR*^19del^, *EGFR*^G724S^, or *EGFR*^19del+G724S^ treated for 72 h with **c** erlotinib or **d** osimertinib. Experiments were performed at least three times. **e**, **f** Graphs show tumor volume of mice injected with NIH-3T3 *EGFR*^19del^ and *EGFR*^19del+G724S^ cells treated with osimertinib (5 mg/kg, i.p., once daily). **e** Tumor volumes (*EGFR*^19del^ vehicle, *n *= 7 mice; *EGFR*^19del^ osimertinib, *n* = 8 mice; *EGFR*^19del+G724S^ vehicle, *n* = 7 mice; *EGFR*^19del+G724S^ osimertinib, *n* =10 mice) were assessed for 20 days by longitudinal caliper measurements every second day following treatment initiation. **f** Tumor volumes were quantified after 8 days of treatment. Volume changes in the osimertinib treatment cohort (dark gray and green) were compared with the vehicle-treated control group (light gray and green). Each dot represents a single tumor per mouse. Significance is calculated by two-tailed Student’s *t* test, n.s.: non-significant. **g** Representative images of Cleaved Caspase-3 stainings. Tumors of mice bearing NIH-3T3 *EGFR*^19del^ or *EGFR*^19del+G724S^ cells were treated with vehicle solution HPMC (0, 5%) or osimertinib. Scale bar 100 μm. **h** Quantification of Cleaved Caspase-3 staining. Number of apoptotic cells in the osimertinib-treated cohort (dark gray and green) was compared with the vehicle-treated control group (light gray and green)

To test the functional relevance of the identified *EGFR* mutation, we overexpressed different combinations of *EGFR*^G724S^ and *EGFR*^19del^ mutations in NIH-3T3 cells. Erlotinib as well as osimertinib treatment resulted in a major reduction of phospho-EGFR levels in *EGFR*^19del^-mutant cells already at concentrations of 0.3 µM of osimertinib but not in cells that expressed *EGFR*^G724S^ either alone or in combination with *EGFR*^19del^ (Fig. 2a, b). We observed similar results with the third-generation EGFR inhibitor rociletinib, despite its lower potency against the *EGFR*^19del^-mutant (Supplementary Fig. 2A).

To validate our findings in an independent cellular model we generated Ba/F3 cells that overexpress *EGFR*^19del^ or *EGFR*^G724S^ alone and the combination of these mutations. The survival of these murine cells initially relies on IL-3 but can be switched to an oncogene such as mutant *EGFR*^22,23^. As expected the introduction of *EGFR*^19del^ or *EGFR*^G724S^ alone led to a transformation of Ba/F3 cells, but only *EGFR*^19del^ showed high sensitivity to erlotinib and osimertinib (Fig. 2c, d, Supplementary Fig. 7A, B).

Based on these results and the increasing relevance of osimertinib in the front-line setting we focused on the resistance phenotype against this third-generation EGFR inhibitor^13^. As in the patients we assume the *EGFR*^G724S^ resistance mutations to occur in the background of *EGFR*^19del^ we compared *EGFR*^19del^ and *EGFR*^+G724S^ sensitivity to third-generation EGFR inhibitors in vivo. To this end we employed murine xenograft models in which genetically modified NIH-3T3 cells were injected subcutaneously into nude mice (*NCR*^*nu/nu*^*)*. Again, we observed efficient tumor formation for both double-mutant *EGFR*^19del+G724S^ and single-mutant *EGFR*^19del^ NIH-3T3 cells (Fig. 2e). Confirming our in vitro results osimertinib (5 mg/kg daily) treatment significantly slowed down tumor growth of *EGFR*^19del^ NIH-3T3 cells compared with vehicle-treated tumors (*p* = 0.027). Of note, osimertinib has a favorable pharmacokinetic profile and is known to halt tumor growth in EGFR-dependent patient-derived cell line xenografts at doses as low as 1 mg/kg daily^10^. However, we observed virtually no therapeutic effect for osimertinib (5 mg/kg daily) treated in double-mutant *EGFR*^19del+G724S^ NIH-3T3 cells compared with vehicle-treated tumors (Fig. 2e, f). Since NIH-3T3 cells can partially form tumors in the absence of an oncogenic driver we did not observe any tumor shrinkage in our xenograft model, as one would expect for xenografts implanted with patient-derived cells^24,25^. As expected, we observed a significant induction of cleaved caspase-3-positive cells (*p* = 0.037) (Fig. 2g, h) and a robust reduction of Ki67 positive cells only in *EGFR*^19del^-mutant but not in *EGFR*^19del+G724S^-mutant tumors that received osimertinib (Supplementary Fig. 2B–F).

These results clearly indicate that the *EGFR*^G724S^ point mutation may confer resistance against third-generation EGFR inhibitors.

### Structural impact of an altered glycine-rich loop conformation

The glycine-rich loop is a crucial structural element for substrate and ligand binding. It is a highly conserved flexible element located in the N-lobe of the kinase domain and contains the canonical GxGxxG motif, where x may be any amino acid^26,27^. It does not come as a surprise that mutations in the glycine-rich loop can interfere with ligand binding and thus mediate resistance to kinase inhibitors as it was described previously for chronic myelogenous leukemia, where mutations in the glycine-rich loop in BCR-ABL cause resistance to imatinib^28,29^.

**Fig. 3:**
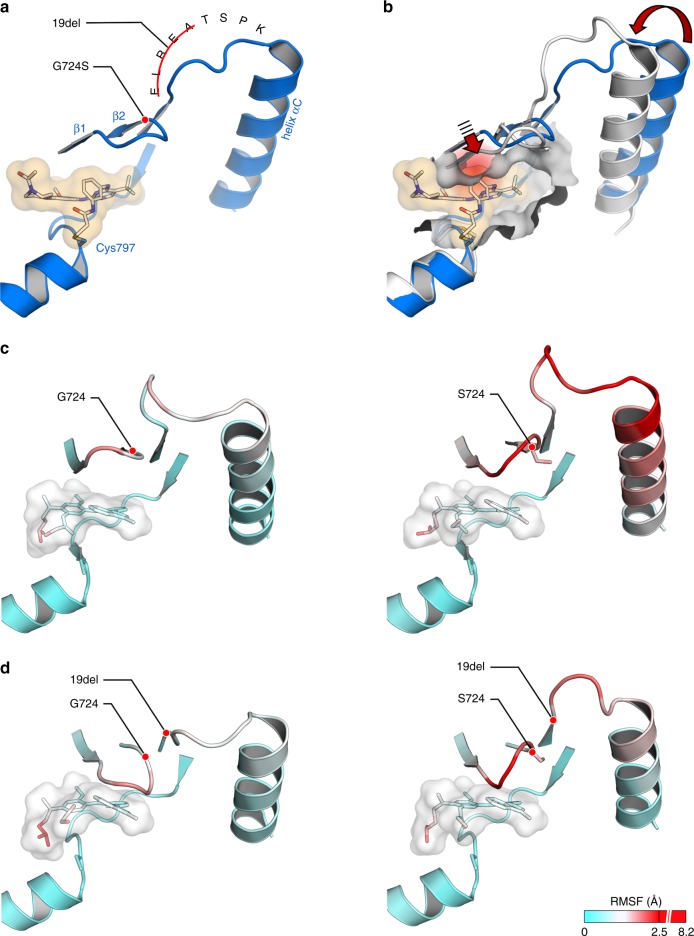
Structural analysis of EGFR^19del+G724S^. **a** Binding site of rociletinib bound EGFR (PDB ID: 5UWD). **b** Alignment of the rociletinib bound EGFR-binding site (blue, PDB ID: 5UWD) with exon 20-mutant *EGFR* (white, PDB ID: 4LRM) that reveals steric hindrance of third-generation TKIs with the glycine-rich loop and the adjacent sheet β1 upon a perturbed network between helix αC, ELREA motif, and glycine-rich loop. **c** Comparison of MD simulations of osimertinib bound EGFR^WT^ (left), EGFR^G724S^ (right) (based on PDB ID: 4ZAU). **d** Comparison of MD simulations of osimertinib bound EGFR^19del^ (left) or EGFR^19del+G724S^ (right) (based on PDB ID: 4ZAU)

To assess the structural impact of the *EGFR*^G724S^ mutation on the EGFR kinase we performed structural analysis based on a previously published co-crystal structure of rociletinib bound to EGFR (PDB ID: 5UWD) (Fig. 3a)^30^. As described before the glycine-rich loop is an essential element for ligand binding and the glycine at position 724 is in direct contact with the adjacent ELREA motif that is subject to deletion mutations in affected patients. The ELREA sequence plays a crucial role in the alignment of the regulatory helix αC that is a key element in the transition between the active and inactive kinase domain conformations^30^. It is therefore conceivable that the *EGFR*^G724S^ mutation influences structure and dynamics of the binding site and thereby the affinity toward third-generation EGFR inhibitors. To illustrate the resistance mutation on the molecular level, we performed an alignment of a third-generation TKI bound to the EGFR-binding site (PDB ID: 5UWD) with a crystal structure of an exon 20-mutated form of *EGFR* (PDB ID: 4LRM). The experimental structure determination of the exon 20 mutant reveals a perturbed network of interactions within the regulatory important helix αC, the adjacent ELREA motif and the glycine-rich loop^31,32^, which we believe to be similar to the investigated G724S mutant. The alignment of these structures suggests that the glycine-rich loop can exist in a conformation that is incompatible with third-generation inhibitor binding (Fig. 3b). Therein, steric repulsion arises from the acrylamide-linker of rociletinib or the methylindole moiety of osimertinib with the sheets β1 and β2 adjacent to the G-rich loop. Although the glycine-rich loop may undergo conformational changes upon ligand binding, the rearrangement might be hindered in the case of third-generation TKIs.

In line with these considerations, molecular dynamics (MD) simulation (based on PDB ID: 4ZAU)^33^ revealed an altered ELREA motif in EGFR^G724S^, as compared with simulated wild-type protein when bound to osimertinib (Fig. 3c, Supplementary Fig. 3A left). Moreover, the introduction of serine to position 724 induces a high degree of dynamic flexibility in the network formed by helix αC, ELREA motif, and glycine-rich loop, as represented by the determined root mean squared fluctuation (RMSF) values (Fig. 3c, Supplementary Fig. 3B; raw data are reported in the B-factor column of PDB structures for osimertinib bound to the EGFR variants studied in Supplementary Data 1–4). These enhanced fluctuations extend toward the methylindole residue of osimertinib. As pointed out in seminal work by Kuriyan, Shaw and co-workers^34^, substantial conformational impact of the exon 19 deletion on the helix αC can be expected, whereas the structural and dynamical influence of additionally introducing the G724S mutation is unknown. Hence, we additionally simulated the corresponding EGFR^19del^ and EGFR^19del+G724S^ systems **(**Fig. 3d, Supplementary Fig. 3A right). Remarkably, despite the strain introduced by deleting the ELREA motif, increased flexibility particularly of the G-rich loop within the regulatory network is also evident for EGFR^19del+G724S^ relative to EGFR^19del^. This finding is robust with respect to varying the starting structures of the simulations (Supplementary Fig. 3B). It appears that the mutant Ser724 side chain renders the regulatory network more flexible and induces altered conformations to the G-rich loop.

Based on these findings, a second line of argumentation could be valid: rather than steric repulsion, the increased flexibility might result in the loss of important interactions between third-generation inhibitors and the binding site that lead to the observed drug resistance. Taken together, we conclude that the *EGFR*^G724S^ mutation may provoke a conformation of the glycine-rich loop, which is incompatible with ligand binding and accounts for decreased binding efficiency as determined for third-generation EGFR inhibitors.

### Altered EGFR inhibitor activity pattern through *EGFR*^G724S^

**Fig. 4:**
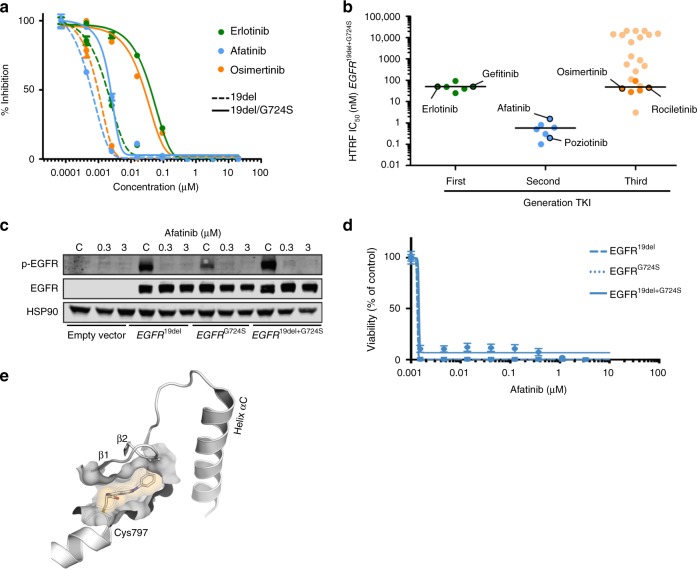
Biochemical profiling of EGFR^G724S^. **a** Homogeneous time-resolved fluorescence (HTRF) is used for IC_50_-determination for EGFR^19del^ and EGFR^19del^^+G724S^ with representative inhibitors. Representative dose–response curves of a single measurement in duplicates are shown. **b** Comparison of biochemical IC_50_-values with HTRF for the three generations of EGFR TKIs against EGFR^19del+G724S^. Values are the mean of three independent measurements in duplicates. **c** Immunoblotting results of NIH-3T3 cells (empty vector, EGFR^19del^, EGFR^G724S^ or EGFR^19del+G724S^) showing phospho-EGFR and total EGFR under afatinib treatment (24 h). HSP90 was used as loading control (*n* = 3). **d** Dose–response measurement of Ba/F3 cells expressing EGFR^19del^, EGFR^G724S^, or EGFR^19del+G724S^ treated for 72 h with afatinib. Experiments were accomplished for at least three times. **e** Structure of exon 20 mutant *EGFR*, bound to 4-anilinoquinazoline based TKI PD168393, is shown

We next addressed the question whether the *EGFR*^G724S^ mutation might directly interfere with the ability of third-generation EGFR inhibitors to bind to the EGFR kinase, as the mutation site is located in the glycine-rich loop, which is an important regulatory element (Supplementary Fig. 4A). Similar to our observations in NIH-3T3 cells, we detected a marked increase of IC_50_-values in kinase assays using osimertinib against the double-mutant EGFR^19del+G724S^ as compared with the EGFR^19del^ single-mutant protein (100-fold) (Fig. 4a).

Having established the kinase assay platform, we next sought to test whether the *EGFR*^G724S^ mutation induces resistance against a specific class of EGFR inhibitors. Herein, we collected a library of more than 120 compounds, of which 90 compounds are proprietary and hence the results of 32 readily published compounds with a known anti-EGFR profile covering clinically relevant first-, second-, and third-generation EGFR inhibitors are discussed in the following (Fig. 4b, Supplementary Fig. 4B, Supplementary Table 2). We screened these inhibitors against both the single and the double-mutant EGFR kinase and observed two interesting patterns of inhibitor activity: (i) the introduction of the *EGFR*^G724S^ mutation in addition to the *EGFR*^19del^ mutation induces resistance against virtually all clinically used first- and third-generation inhibitors and (ii) all second-generation inhibitors including afatinib, poziotinib, and dacomitinib remained active against the EGFR^19del+G724S^ double-mutant kinase (Fig. 4b, Supplementary Table 2). Although, first- and second-generation inhibitors exhibit the same quinazoline scaffold, a remarkable difference in biochemical potency is evident. Alkylation of Cys797 is a distinct feature of second-generation TKIs that discriminates them from first-generation inhibitors. This finding indicates that a covalent bond formation to the target kinase is crucial to occupy the binding site efficiently. Interestingly, we also identified an aminoindazole-based inhibitor with low nanomolar activity against the double-mutant kinase that would not be classified as a second-generation EGFR inhibitor but indeed exhibits a different binding mode than osimertinib (Fig. 4b, Supplementary Table 2)^35^.

We next tested whether the biochemical activity of second-generation EGFR inhibitors in *EGFR*^G724S^-mutant cells translates into a therapeutically relevant on-target activity in cellular models. We therefore assessed phospho-EGFR levels following afatinib exposure in NIH-3T3 cells expressing either the empty vector or vectors with *EGFR*^19del^, *EGFR*^G724S^ alone, or the combination of both mutations *EGFR*^19del+G724S^ (Fig. 4c, Supplementary Fig. 7C). As expected, afatinib treatment led to a reduction of phospho-EGFR signaling that was independent of the presence of the *EGFR*^G724S^ mutation in the glycine-rich loop at concentrations between 10–100 nM (Fig. 4c, Supplementary Fig. 4C). We validated these findings in our Ba/F3 cell lines and found that *EGFR*^G724S^-mutant cells largely retained sensitivity to afatinib at low nanomolar concentrations (Fig. 4d).

These results triggered us to revisit our previously analyzed crystal structure of exon 20-mutant *EGFR* (PDB ID: 4LRM) (Supplementary Fig. 5). In line with our biochemical data this structure shows that the binding of second-generation TKIs based on the 4-anilinoquinazoline scaffold (PD168393) to the altered binding site is well tolerated (Fig. 4e).

Thus, our data indicate that the *EGFR*^G724S^ mutation induces resistance toward third- and first-generation but retains sensitivity toward 4-aminoquinazoline based second-generation EGFR inhibitors.

### *EGFR*^G724S^ reduces binding of third-generation EGFR TKI

**Fig. 5:**
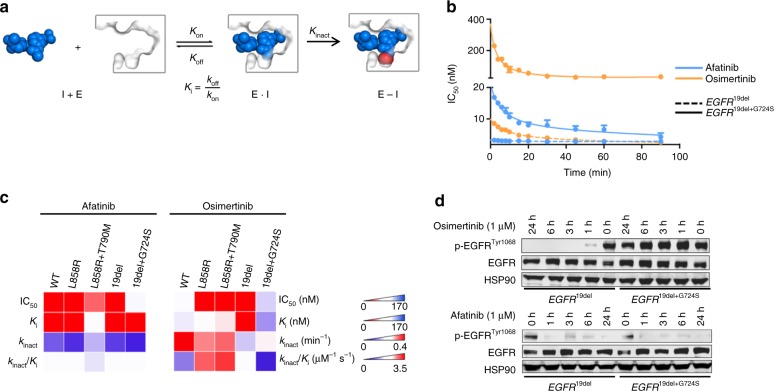
Kinetic evaluation of second- and third-generation EGFR TKIs against EGFR mutant proteins. **a** Schematic overview of two-step binding mechanism of covalent inhibitors to kinases with *K*_i_ (quotient of *k*_off_ and *k*_on_) describing the reversible binding affinity and *k*_inact_ describing the rate of inactivation. **b** Time-dependent IC_50_-determination of afatinib and osimertinib on EGFR mutant proteins. Representative curves of single measurements in duplicates are shown. **c** Heatmap of biochemical IC_50_-, *K*_i_-, and *k*_inact_ determination of second- and third-generation EGFR TKIs against EGFR mutant proteins. Values are the mean of three independent measurements in duplicates. **d** Immunoblotting results of NIH-3T3 cells (EGFR^19del^ or EGFR^19del+G724S^) monitoring phospho-EGFR and total EGFR. Cells were treated for indicated times (0, 1, 3, 6, and 24 h) with osimertinib (1 µM) or afatinib (1 µM). HSP90 was used as loading control (*n* = 3)

Further in-depth kinetic evaluation including determination of kinetic parameters *K*_i_ and *k*_inact_ was conducted to more accurately define differences between second- and third-generation inhibitor binding. Binding of covalent inhibitors to a kinase is assumed to succeed in a two-step process: first the inhibitor binds to the kinase in a reversible fashion characterized by *K*_i_ and in a second step the covalent bond is formed which can be specified with the rate of inactivation (*k*_inact_) (Fig. 5a)^36,37^.

In these experiments, we identified marked differences in the binding characteristics of second- and third-generation EGFR inhibitors to *EGFR-*mutants (Fig. 5b, c, Supplementary Table 3). For instance, the *k*_inact_ of osimertinib and rociletinib appears to be similar among the *EGFR*-mutants and the *EGFR*^G724S^ mutation does not negatively affect the covalent bond formation with Cys797. Our data further indicate that the *EGFR*^G724S^ mutation has a strong impact on the formation of the reversible protein–ligand complex in the context of these drugs which prove to bind in a less-affine manner indicated by increased *K*_i_-values (osimertinib *EGFR*^19del^ < 1 nM and *EGFR*^19del+G724S^ 80 nM) (Fig. 5b, c). The second-generation EGFR inhibitor afatinib and the structurally related inhibitor poziotinib exhibit constant affinities and binding kinetics for EGFR^WT^, EGFR^19del^, and EGFR^19del+G724S^ kinases (Fig. 5b, c). In addition, our data reveal that the loss of *EGFR*^T790M^, as observed in all of the relapsed tumors that were enriched for *EGFR*^G724S^-positive clones, further enhanced the loss of affinity of third-generation EGFR inhibitors. Third-generation inhibitors are designed to target a methionine gatekeeper residue in position 790, whereas second-generation inhibitors afatinib and poziotinib exhibit a more pronounced affinity toward threonine-carrying EGFR variants. Based on these findings we hypothesized that such a marked difference between afatinib and osimertinib in the engagement of the mutant kinase should be also detectable in cellular assays. We therefore tested the ability of afatinib and osimertinib to inhibit phospho-EGFR over time (Fig. 5d, Supplementary Fig. 7D, E). In line with our biochemical data even at 1 µM concentrations osimertinib was not able to reduce phospho-EGFR levels in NIH-3T3 cells expressing EGFR^19del+G724S^. In contrast to that, afatinib depleted phospho-EGFR levels in these cells as efficiently as in EGFR^19del^ cells (Fig. 5d, Supplementary Fig. 7D, E).

Thus, our data suggest that in EGFR^G724S^-mutant kinase the reversible binding of second-generation EGFR inhibitors is superior to third-generation EGFR inhibitors and might therefore overcome *EGFR*^G724S^-driven resistance.

### *EGFR*^G724S^ is sensitive to second-generation EGFR inhibitors

**Fig. 6:**
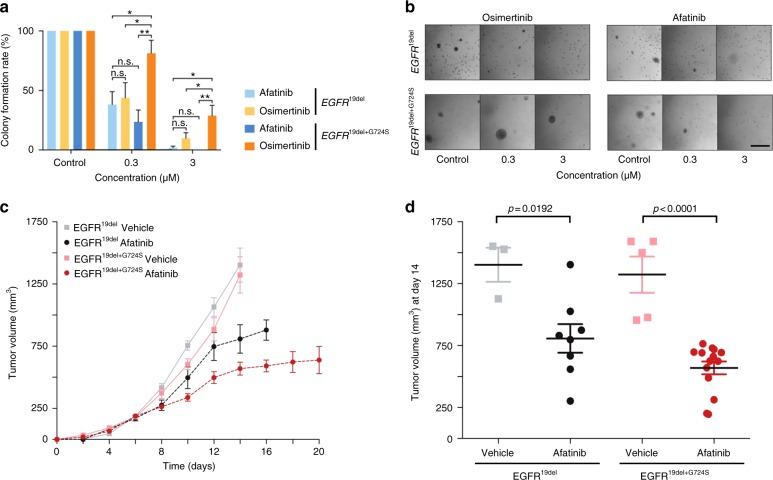
Sensitivity of *EGFR*^G724S^ to second-generation EGFR inhibitor in vitro and in vivo. **a** Relative mean colony number of NIH-3T3 *EGFR*^19del^ cells compared with *EGFR*^19del+G724S^ cells was assessed in soft agar assays after 16 days under treatment. Cells were treated with osimertinib or afatinib. **b** Representative images of colonies under treatment are displayed in the lower panel. Scale bars, 50 μm (*n* = 4), n.s.: non-significant, * = *p* < 0.05, ** = *p* < 0.01. **c**, **d** Graphs show tumor volume in mice injected with NIH-3T3 *EGFR*^19del^ and *EGFR*^19del+G724S^ cells treated with afatinib (20 mg/kg, p.o., once daily). **c** Tumor volumes (*EGFR*^19del^ vehicle, *n* = 13 mice; *EGFR*^19del^ osimertinib, *n *= 8 mice; *EGFR*^19del+G724S^ vehicle, *n* = 11 mice; *EGFR*^19del+G724S^ osimertinib, *n* =14 mice) were assessed for 20 days by longitudinal caliper measurements every second day following treatment initiation. **d** Tumor volumes were quantified after 8 days of treatment. Volume changes in the afatinib treatment cohort (dark gray and red) were compared with the vehicle-treated control group (light gray and light red). Each dot represents one tumor from a mouse. Significance is indicated by asterisk and calculated by two-tailed Student’s *t* test

A previous case report of a patient with acquired *EGFR*^G724S^ mutation showed a remarkable response to the combination of osimertinib and afatinib^21^. To test whether the observed affinity to second-generation EGFR inhibitors translates into cellular activity we tested the ability of afatinib as single agent to outperform osimertinib activity in *EGFR*^19del+G724S^-driven cells. To this end, we plated NIH-3T3 cells that express either EGFR^19del^ or EGFR^19del+G724S^ in soft agar and treated the cells with increasing concentrations of both drugs over the time of 2 weeks. In line with our in vivo results osimertinib was only effective against the formation of *EGFR*^19del^-driven colonies at submicromolar concentrations but not against *EGFR*^19del+G724S^-mutant cells (Fig. 6a, b). However, afatinib largely prevented outgrowth of both *EGFR*^19del^ and *EGFR*^19del+G724S^-driven colonies at submicromolar concentrations. Thus, the growth inhibition effect of afatinib compared with osimertinib was significantly higher (*p* = 0.01) in *EGFR*^19del+G724S^-mutant cells (Fig. 6a, b).

To further evaluate the sensitivity of *EGFR*^G724S^ either alone or in combination with *EGFR*^19del^ to third-generation EGFR inhibitors in vivo we employed our murine xenograft models. Again, both single-mutant *EGFR*^19del^ and double-mutant *EGFR*^19del+G724S^ NIH-3T3 cells formed fast growing tumors in nude mice (Fig. 6c). As expected, afatinib (20 mg/kg daily) treatment significantly slowed down tumor growth of both single-mutant *EGFR*^19del^ (*p* = 0.0192) and double-mutant *EGFR*^19del+G724S^ (*p* < 0.0001) xenograft when compared with vehicle-treated tumors (Fig. 6d, Supplementary Fig. 6).

Overall, our results indicate that acquired *EGFR*^G724S^ mutations robustly limit the activity of third-generation inhibitors, whereas sparing the second-generation EGFR inhibitors such as afatinib. Existing Food and Drug Administration (FDA)-approvals for some of these drugs may facilitate the translation of our results into clinical practice for genetically defined osimertinib-resistant lung cancer patients.

## Discussion

EGFR inhibitors represent a showcase for the therapeutic power of precision cancer medicine in genetically selected patients. Building on structural and functional insights, several lines of drug development efforts provided a rich source of clinically available drugs including second-generation EGFR inhibitors^38–40^. So far, these drugs played only a minor role for the targeted treatment of *EGFR*-mutant tumors as they largely failed to induce pronounced effects in patients that acquired a gatekeeper *EGFR*^T790M^ mutation^22,41,42^. Although second-generation EGFR inhibitors bind irreversibly to EGFR these drugs lack the flexibility to circumvent a steric clash with the gatekeeper mutation^22^. Second-generation EGFR inhibitors are also very potent inhibitors of *EGFR*^WT^ and therefore these drugs are likely to induce diarrhea or skin rash^41^. At the same time, afatinib is an FDA-approved drug for first-line treatment of *EGFR-*mutant LADC with a known on-target resistance profile derived from preclinical models^43,44^.

Our clinical and functional characterization of the acquired *EGFR*^G724S^ resistance mutation suggests a revision of the properties and clinical liabilities that are associated with second-generation EGFR inhibitors. This is becoming an even more relevant issue in the light of the encouraging results achieved with osimertinib in the front-line setting that might challenge the standard use of first-generation EGFR inhibitors in *EGFR* mutant patients^13^. First of all, the emergence of an *EGFR*^G724S^ resistance mutation is associated with loss of *EGFR*^T790M^-positive subclones, and thus providing the right genetic context for the use of second-generation EGFR inhibitors. These observations are in line with previous reports on acquired osimertinib resistance that frequently report a loss of *EGFR*^T790M^ -positive clones^15,16^. Although we cannot exclude the fact that additional signaling layers including FAK or SFK or co-occuring mutations that were not part of our panel may be involved in the observed resistance phenotype^45–47^, our genomic and functional data strongly support a dominant role of the acquired *EGFR*^G724S^ mutation in the context of third-generation EGFR inhibitors. Thus, our findings might be relevant for a large group of patients receiving third-generation inhibitors. Interestingly, previous studies that characterized resistance patterns to third-generation EGFR inhibitors did not capture recurrent *EGFR*^G724S^ mutations^14,48^. Although our limited sample size precludes broad conclusion on the real prevalence of this mutation it is conceivable that the size of the previous studies or a potential compound selection bias (osimertinib vs. rociletinib) may have underestimated the relevance of *EGFR*^G724S^ mutations as a potential resistance mechanism. Our data indicate that *EGFR*^G724S^ mutations seem to primarily affect the reversible first step of third-generation inhibitor binding within the ATP-binding pocket before irreversible attachment to Cys797 can occur. We conclude that the observed fluctuation of the glycine-rich loop plays a role in this phenotype, similar to other systems where anti-correlations between flexibility and ligand-binding affinity have been observed^49,50^. However, although the kinetics of third-generation EGFR inhibitor binding are perturbed dramatically, second-generation EGFR inhibitors are potent enough to establish such a reversible binding despite the *EGFR*^G724S^ mutation. Thus, their liability in terms of an efficient engagement of *EGFR*^WT^, in the context of *EGFR*^G724S^ resistance mutations turns into an asset.

Given the increasing number of patients receiving osimertinib our data are of high clinical relevance. Importantly, our study provides a molecular basis for the ability of *EGFR*^G724S^ to induce resistance and suggest that second-generation EGFR inhibitors might overcome osimertinib resistance in these patients.

## Methods

### Experimental design

The aim of this study was to examine the effects of the acquired *EGFR*^G724S^ mutation that was observed in lung tumors that become resistant to osimertinib treatment. We conducted systematic cellular, in vivo, biochemical, and structural analyses to determine the functional relevance of this mutation in the context of first-, second-, and third-generation EGFR inhibition. To investigate the *EGFR*^G724S^ mutation in combination with the different EGFR inhibitors we used the mouse fibroblast cell line NIH-3T3 and female nude mice. All experiments including immunoblotting, tumor volume measurement, soft agar assays, and biochemical assays were performed at least three times. For the biochemical analysis, we used activity-based assay for IC_50_-determination and kinetic characterization. Each reaction was performed in duplicate, and at least three independent determinations of each IC_50_ were made. To characterize the acquired *EGFR*^G724S^ resistance mutation on a molecular level we used structural modeling of EGFR kinase and validated our observations using computational modeling of publically available co-crystal structures. For detailed information please see “Methods”. The local animal protection committee and the local authorities approved all animal procedures. All patients consented into the analyses according to the local practice.

### Patients, efficacy assessments, and sample collection

The four patients included into this analysis were treated with the third-generation EGFR inhibitor osimertinib within the AURA trial (NCT01802632), the compassionate use program or clinical routine upon progression to EGFR-targeted therapy (P1–P4). One patient, P2, also received treatment with the third-generation EGFR inhibitor EGF816 within the CEGF816X2101 phase I trial (NCT02108964). All patients consented to treatment according to the good clinical practice guideline and were treated according to the trial protocol and/or local practice. Patients received osimertinib at a dose of 80 mg qd and were treated until progression. Treatment doses were adapted if necessary in case of toxicity and adverse events. Tumor assessment was performed by computed tomography (CT) or ^18^FDG PET and magnetic resonance imaging according to the specifications given in the trial protocols and/or according to local standards. Efficacy was assessed using the response evaluation criteria for solid tumors, version 1.1 in patients P1 and P3 (RECIST 1.1)^51^. In patients P2 and P4 RECIST evaluation was not feasible. Response to osimertinib treatment was performed by ^18^FDG PET-CT in P2. In P4 baseline CT scan was older than 4 weeks, not fulfilling the requirements set up by RECIST 1.1. In patients where RECIST evaluation was not possible, progression was defined by the treating physician as growth of clinically significant lesions. Biopsy collection was performed through core needle biopsy, excisional biopsy, or cytology according to local standard procedures at time points T0 to T3. Samples at time points T1, T2, and T3 were collected in progressing tumor lesions. All tumor samples were fixed in formalin (4%) and embedded in paraffin (FFPE). To assess the frequency of *EGFR*^G724S^ in the setting of acquired resistance to third-generation EGFR inhibitors, we analyzed FFPE tissue of patients from the Network Genomic Medicine and collaborating institutions. *EGFR*-mutant NSCLC patients who fulfilled the following criteria were included into the analysis: (1) sufficient tumor tissue for genomic characterization, (2) progressive disease while on treatment with a third-generation EGFR inhibitor. All patients consented into the analyses according to the local practice.

### Targeted next-generation sequencing

Tumor tissue of patients was genomically characterized by massively parallel sequencing (MPS) and fluorescence in situ hybridization (FISH), if feasible. Until March 2015, MPS was carried out with an Ion AmpliSeq Custom DNA Panel (Thermo Fisher Scientific, Waltham, MA, USA) (Lun3 panel) and a MiSeq benchtop sequencer (Illumina, San Diego, USA). As from March 2015 MPS was carried out with a GeneRead DNAseq Custom Panel V2 (Qiagen, Hilden, Germany) consisting of 205 amplicons (Lun4 panel). Library preparation was performed according to the GeneRead DNAseq Gene Panel Handbook (Qiagen) as described earlier^52^.

### Cell viability

In all, 5000 Ba/F3 cells/well were seeded in triplicates in a white-bottom 96-well plate in 90 μl media/well. Compounds were prepared by serial dilution. Dimethyl sulphoxide was added to control wells in the highest dilution used in the assay. The cells were treated for 72 h with the compounds following determination of ATP content as surrogate for viability by CellTiter-Glo® assay (CTG) (Promega). CTG was incubated for at least 20 min on the cells without light. Luminiscence was assessed on an Infinite 200 Pro microplate reader (Tecan). Data were analyzed and plotted in PRISM.

### Cell culture

NIH-3T3 cells were cultured in Dulbecco's Modified Eagle's medium (DMEM) with 10% fetal bovine serum (FBS) (Thermo Fisher Scientific) and 1% penicillin–streptomycin (Thermo Fisher Scientific). Ba/F3 cells were cultured in Roswell Park Memorial Institute with 20% FBS and 1% penicillin–streptomycin (Thermo Fisher Scientific). The cells were incubated at 37 °C and 5% CO2 in a humidified incubator. Cell lines expressing recombinant EGFR variants were generated by retroviral transduction. In brief, cDNA sequences encoding EGFR^E746-S752del^, EGFR^G724S^, or EGFR^E746-S752del+G724S^ were cloned into a pBabe-puro vector and co-transfected with pCL-Eco helper plasmid into HEK 293T cells using TransIT-LT1 reagent (Mirus). After 48 h of transfection, retroviral particles were collected for infection of NIH-3T3 cells and Ba/F3 cells. After 24 h of infection, medium was replenished with growth medium containing puromycin (3 µg/ml) to select for transduced cell clones. Cells were treated with first-, second-, and third-generation EGFR inhibitors (erlotinib, osimertinib, rociletinib (Selleckchem), and afatinib (LC Laboratories)) with different concentrations. Ba/F3 cells were a kind gift from Nikolas von Bubnoff. NIH-3T3 cells were purchased from the “Deutsche Sammlung von Mikroorganismen und Zellkulturen (DSMZ) and the HEK 293T were purchased from the American Type Culture Collection (ATCC)”. All cell lines were authenticated with the STR method and were tested negative for mycoplasma contamination by qPCR analyses from GATC Biotech services.

### Soft agar assays

On a layer of 1% bottom agar (Sigma-Aldrich) 10000 NIH-3T3 cells per well of a 12-well plate were suspended in DMEM (Thermo Fisher Scientific) containing 0.6% agar, 10% calf serum (PAA Laboratories), 0.5% sodium bicarbonate (PAN Biotech) and 1% sodium pyruvate (Thermo Fisher Scientific). After 16 days incubation pictures were taken with a Zeiss Axiovert 40 CFL microscope at × 100 magnification and colony size was assessed with ImageJ (http://rsbweb.nih.gov/ij/).

### Immunoblotting

For immunoblot analysis, cells were harvested and lysed in cold lysis buffer in the presence of protease and phosphatase inhibitors (Cell Signaling). Equal amounts (20 µg) of protein were separated on 4–20% Novex Tris-Glycine gels (Invitrogen), blotted on polyvinylidene difluoride membranes and incubated with specific primary antibodies and fluorescently labeled secondary antibodies (IRDye, LI-COR). Proteins were detected with the Odyssey CLx imaging system (LI-COR). Protein levels were quantified with ImageStudio (LI-COR) and normalized to loading control. The following primary antibodies were used: total EGFR (Cell Signaling #2232, dilution 1:1000), HSP90 (Cell Signaling #4877, dilution 1:1000), p-EGFR Tyr1068 (Invitrogen #36-9700, dilution 1:1000). Anti-rabbit IgG (Cell Signaling #5151, 1:10000) was used as secondary antibody. Uncropped raw blots corresponding to data in Figs. 2a, 2b, 4c, and 5d can be found in the Supplementary Information.

### Xenograft models

The local animal protection committee and the local authorities approved all animal procedures. Osimertinib (Cayman Chemical) was dissolved in 0.5% (Hydroxypropyl-) methylcellulose (Sigma-Aldrich) to a final concentration of 20 mg/ml. Osimertinib was administered daily up to 12 days at a dose of 5 mg/kg and afatinib at a dose of 20 mg/kg by oral gavage. NIH-3T3 *EGFR*^19del^ and NIH-3T3 *EGFR*^19del+G724S^ cells were resuspended in serum-free DMEM medium with 1% penicillin–streptomycin (Thermo Fisher Scientific) (at a concentration of 2 × 10^6^ cells in 100 μl) and injected subcutaneously in both flanks of 8–12 weeks old female nude mice (RJ:NMRI-FOXN1 NU, Janvier Labs). Upon formation of palpable subcutaneous tumors (200–300 mm^3^ tumor volume), mice were treated with vehicle solution (Hydroxypropyl-) methylcellulose (0.5%) or with osimertinib. Tumor size was monitored every second day by measurement of perpendicular diameters by an external caliper and calculated by use of the modified ellipsoid formula: *V* = 0.5×(length×width^2^). Mice were killed and subcutaneous tumors were resected and fixed in 4% formalin for 24 h and embedded in paraffin. The harvested tumor samples were stained against the apoptotic-marker Cleaved Caspase-3, and the proliferation marker Ki67. For a quantification purpose, each marker was quantified using ten high-power-field (× 400) pictures and the median was calculated for the given marker.

### Immunohistochemical staining

Tissue samples were incubated in 4% formalin overnight and subsequently embedded in paraffin. For tissue analysis, 3–5 μm sections were cut, de-paraffinized, and antigen retrieval was performed using either citrate at pH 6.0, or ethylenediaminetetraacetic acid (EDTA) at pH 9.0 for 20 min. Washing steps were performed using phosphate-buffered saline. Primary antibodies were purchased from Cell Signaling (Cleaved Caspase-3, #Asp175, dilution 1:100) and Cell Marque (Ki67, #SP6, dilution 1:100). Corresponding secondary antibody detection kits were used from Histofine® Simple stain and stained on an automated stainer (LabVision Autostainer 480S; Thermo Fisher).

### Computational modeling

The structure 4ZAU deposited in the PDB was used as basic template for modeling the noncovalently bound EGFR-osimertinib complex. Missing residues were obtained from PDB entries 5CZH^53^ for residues 748–755 (LREATSPKA/LREATSPKA), 863–865 (GAE/GAE), 873–874 (GG/GG), 985–991 (ERMHLP/ERMHLP), 1003–1007 (DEEDM/DEEDM) and from 3PP0^54^ for residues 748–755 (LREATSPKA/LRENTSPKA), 863–865 (GAE/GAE), 874 (G/G), 991–1001 (SPTDSNFYRAL/PLDSTFYRSLL). Terminal regions 693–697 and 1018–1022 were truncated, the mutation G724S and the still missing residue 1002 were introduced by Modeller 9.14^55^. For MD simulations, the proteins and ligand were treated by the ff14SB force field^56^ and the GAFF model^57^, respectively, within AMBER 14^58^. The resulting simulation system for the wild type consisted of 24,358 TIP3P water molecules, seven sodium cations^59^, 5158 protein atoms, and 72 ligand atoms. The G724S system was composed of 25,651 TIP3P molecules, 7 sodium cations, 5162 protein atoms, and 72 ligand atoms. For both, *EGFR*^WT^ and *EGFR*^G724S^ mutation, the same simulation protocol was used, starting with a geometry optimization down to a final RMS gradient of 0.0001 kcal mol^−1^ Å^−1^ followed by 4 ns heating to 298.15 K in the canonical ensemble (Langevin thermostat) while applying harmonic restraints on protein Cα atoms. The resulting system was then simulated over 4 ns in the isothermal–isobaric ensemble (Berendsen barostat) at 1 bar pressure, also under der action of restraints. Finally, restraints except for fixed hydrogen bond distances were removed and the systems were run over 200 ns with a 2 fs time step using AMBER 16^58^. The stability of the simulations systems was checked by computing the structural root mean square deviations (RMSD) of Cα atoms from the respective initial snapshots of the production runs over time. (Supplementary Fig. 3C). The final 100 ns were used for clustering structures taken every 10 ps using the DBSCAN algorithm^60^ in AMBER 16 with distance cutoffs 1.18 Å (*EGFR*^WT^) and 1.205 Å (*EGFR*^G724S^) and a minimum number of points to form a cluster set to 5. Final structures were obtained from centroids of the maximally populated clusters by geometry optimization in an implicit water environment (ALPB)^61^. RMSF fluctuations were computed over the final 100 ns and mapped onto the resulting structures (Supplementary Data 1–4) for further analysis. These structures were then modified by Modeller 9.14^55^ to generate starting models for the simulation of *EGFR*^19del^ and *EGFR*^19del+G724S^ complexes by deleting residues 746 to 750 followed by system preparation steps as before, yielding 24271 water molecules, six sodium ions, 5075 protein- and 72 ligand atoms for *EGFR*^19del^ and 26,866 water molecules, six sodium ions, 5079 protein- and 72 ligand atoms *EGFR*^19del+G724S^. Trajectories were generated and analyzed as before, using the last 75 ns (see RMSD plot Supplementary Fig. 3A) for clustering (DBSCAN cutoffs of 1.16 Å for *EGFR*^19del^ and *EGFR*^19del+G724S^) and RMSF calculations. The simulations of the pure deletion and the double-mutant complexes were performed in order to check the independence of the system stability and fluctuation analysis of the initial conditions.

### Activity-based assay and kinetic characterization

For biochemical assays *EGFR*^WT^, *EGFR*^L858R^, and *EGFR*^L858R+T790M^ were purchased from Carna Bioscience (lot13CBS-0005K for *EGFR*^WT^; Carna, lot13CBS-0537B for *EGFR*^L858R^; and Carna, lot12CBS-0765B for *EGFR*^L858R+T790M^). However, EGFR^19del^ and EGFR^19del+G724S^ were expressed and purified as follows. First DNA-encoding residues compromising the juxtamembrane segment, the kinase domain and the C-terminal tail of human EGFR (UniProt entry P00533, residues 695–1210) were synthesized (GeneArt, Life Technologies). The construct was cloned into pIEX/Bac3 expression vector (MerckMillipore), using BamHI and Bsu36I restriction sites. Mutations were introduced by side-directed mutagenesis (QuikChange, Stratagene/Agilent Technologies). Transfection, virus generation, and amplification were carried out in *Spodoptera frugiperda* cell line *Sf*9 following the BacMagic protocol.

After three days of expression (27 °C, 110 rpm) the cells were harvested (3000 × *g*, 20 min), resuspended in buffer A (50 mM Tris, 500 mM NaCl, 10% glycerol, 1 mM dithiothreitol, pH 8) and homogenized by french press. The lysate was cleared by centrifugation at 40,000 × g for 1 h at 4 °C and loaded on a prepacked column (Glutathione HiCap from Qiagen). The elution was done with a gradient of buffer B (buffer A + 10 mM glutathione). For the final purification step the fractions containing the target protein were combined, concentrated and applied to a HiLoad 16/600 superdex 75 pg column (GE Healthcare) in buffer C (25 mM TRIS, 250 mM NaCl, 10% glycerol, pH 8). The purified protein was concentrated to 5 mg/mL and stored at − 80 °C until further use. Protein identity was confirmed by ESI-MS analysis. IC_50_-determinations for EGFR and its mutants were performed with the HTRF KinEASE-TK assay from Cisbio according to the manufacturer’s instructions. Briefly, the amount of EGFR in each reaction well was set to 0.60 ng of EGFR^WT^ (0.67 nM), 0.10 ng of EGFR^L858R^ (0.11 nM), 0.07 ng of EGFR^L858R+T790M^ (0.08 nM), 1 ng of EGFR^19del^ (1.1 nM) and 0.10 ng of EGFR^19del+G724S^ (0.11 nM). An artificial substrate peptide (TK-substrate from Cisbio) was phosphorylated by EGFR. After completion of the reaction (reaction times: 25 min for WT, 15 min for EGFR^L858R^, 20 min for EGFR^L858R+T790M^, 15 min for EGFR^19del^ and 25 min for EGFR^19del+G724S^), the reaction was stopped by addition of buffer containing EDTA as well as an antiphosphotyrosine antibody labeled with europium cryptate and streptavidin labeled with the fluorophore XL665. FRET between europium cryptate and XL665 was measured after an additional hour of incubation to quantify the phosphorylation of the substrate peptide. An EnVision multimode plate reader (PerkinElmer) was used to measure the fluorescence of the samples at 620 nm (Eu-labeled antibody) and 665 nm (XL665 labeled streptavidin) 50 μs after excitation at 320 nm. The quotient of both intensities for reactions made with eight different inhibitor concentrations was then analyzed using the Quattro Software Suite for IC_50_-determination. Each reaction was performed in duplicate, and at least three independent determinations of each IC_50_ were made. For kinetic characterization (*k*_inact_/*K*_i_), the inhibitors were incubated with *EGFR*-mutants over different periods of time (2−90 min), whereas durations of enzymatic and stop reactions were kept constant as stated above. A sixfold dilution series (eight data points per IC_50_ curve) starting at 20 μM final compound concentrations was applied. Calculated IC_50_-values were plotted versus incubation time, and data were fit as described in the literature to determine *k*_inact_ and *K*_i_^37^. MAb PT66-Eu cryptate (61T66KLB) stock solution was prepared according to manufactures instructions and diluted 1:1 with detection buffer for activity-based assay.

### MET and HER2 FISH analyses

FISH was performed for determination of *MET* gene copy number using ZytoLight SPEC MET/CEN7 Dual Color Probe (ZytoVision). High-level amplification was defined in tumors with (a) *MET/CEN7* ratio ≥ 2.0 or (b) an average *MET* gene copy number per cell of ≥ 6.0, or (c) ≥ 10% of tumor cells containing ≥ 15 *MET* signals. Intermediate level of gene copy number gain being defined as (a) ≥ 50% of cells containing ≥ 5 *MET* signals and (b) criteria for high-level amplification are not fulfilled. Low level of gene copy number gain was defined as (a) ≥ 40% of tumor cells showing ≥ 4 *MET* signals and (b) criteria for high-level amplification or intermediate level of gene copy number gain are not fulfilled. All other tumors were classified as negative. For determination of *HER2* (*ERBB2*) status FISH was performed using a ZytoLight SPEC *ERBB2/CEN17* Dual Color Probe (ZytoVision). Amplification status was classified in analogy to the recommendations of the American Society of Oncology for *HER2* testing in breast cancer. Amplification of *HER2* was positive if (a) *ERBB2/CEP17* ratio ≥ 2.0 or (b) *HER2* GCN ≥ 6.0.

### Statistical analysis

Statistical analysis was performed using GraphPad Prism 5 (GraphPad Software Inc). Data obtained from mice tumor analysis and in vitro assays were subjected to unpaired Student’s *t* test. Data are plotted as means ± standard error of the mean. Quantification of high-power-field analysis was calculated by Mann–Whitney *U* test.

## Electronic supplementary material

Supplementary Information

Description of Additional Supplementary Files

Supplementary Data 1

Supplementary Data 2

Supplementary Data 3

Supplementary Data 4

## Data Availability

The datasets generated during and/or analyzed during the current study are available from the corresponding author on reasonable request.
