# Supplementary material for: Overcoming EGFRG724S-mediated osimertinib resistance through unique binding characteristics of second-generation EGFR inhibitors
Source: Nat Commun. 2018 Nov 7;9:4655. doi: 10.1038/s41467-018-07078-0 (PMC6220297; doi:10.1038/s41467-018-07078-0)
Supplement: Supplementary file 1 — Supplementary Information [file 41467_2018_7078_MOESM1_ESM.pdf]

Overcoming *EGFR*^{G724S}-mediated osimertinib resistance through unique binding characteristics of second-generation EGFR inhibitors

Fassunke et al

A

		1st line	2nd line	3rd line	4th line
**P1**	Treatment	chemotherapy*	erlotinib	osimertinib	-
	ToT (months)	64	22	-	-
	PFS (months)	-	-	8.2	-
**P2**	Treatment	erlotinib	chemotherapy**	EGF816	osimertinib
	ToT (months)	12	29	5.8	7.1
	PFS (months)	-	-	5.6	7.0#
**P3**	Treatment	gefitinib	osimertinib	-	-
	ToT (months)	32	10.1	-	-
	PFS (months)	-	6.8	-	-
**P4**	Treatment	erlotinib	osimertinib	-	-
	ToT (months)	36	4.9	-	-
	PFS (months)	-	2.5#	-	-

B

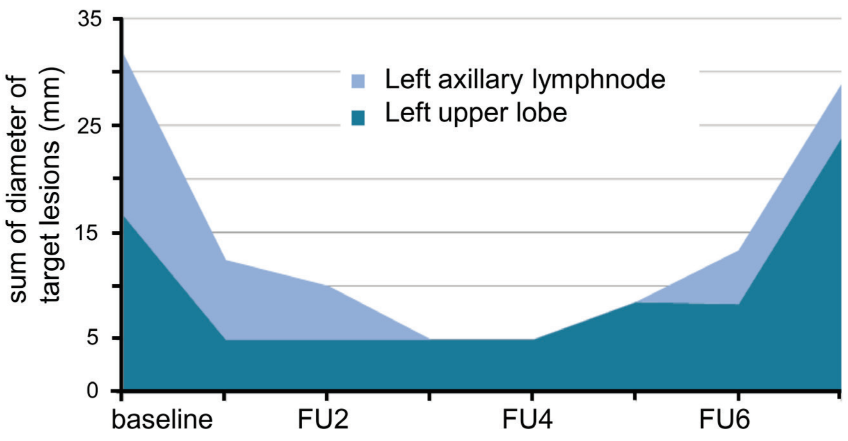

C

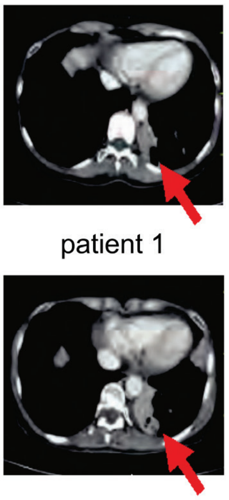

D

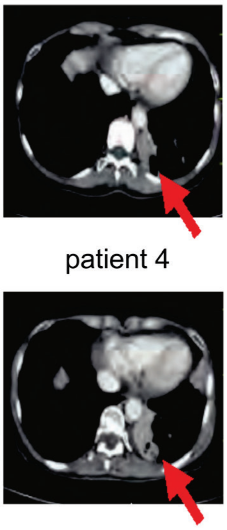

Supplementary Figure 1. Summary of systemic treatments by patient. **A)** Listing of treatment lines, time on treatment and progression-free survival if evaluable. **B)** RECIST analysis of follow-up scans during osimertinib treatment of patient one. Y-axis indicates the sum of the diameter of target-lesions. **C)** and **D)** CT scans prior to treatment with osimertinib (above) and at progression to osimertinib (below) in patients P1 and P4. Follow-up: FU, PFS: progression-free survival, ToT: time on treatment, *adjuvant cisplatin/vinorelbine, **carboplatin/pemetrexed/bevacizumab + maintenance, # PD not determined by RECIST.

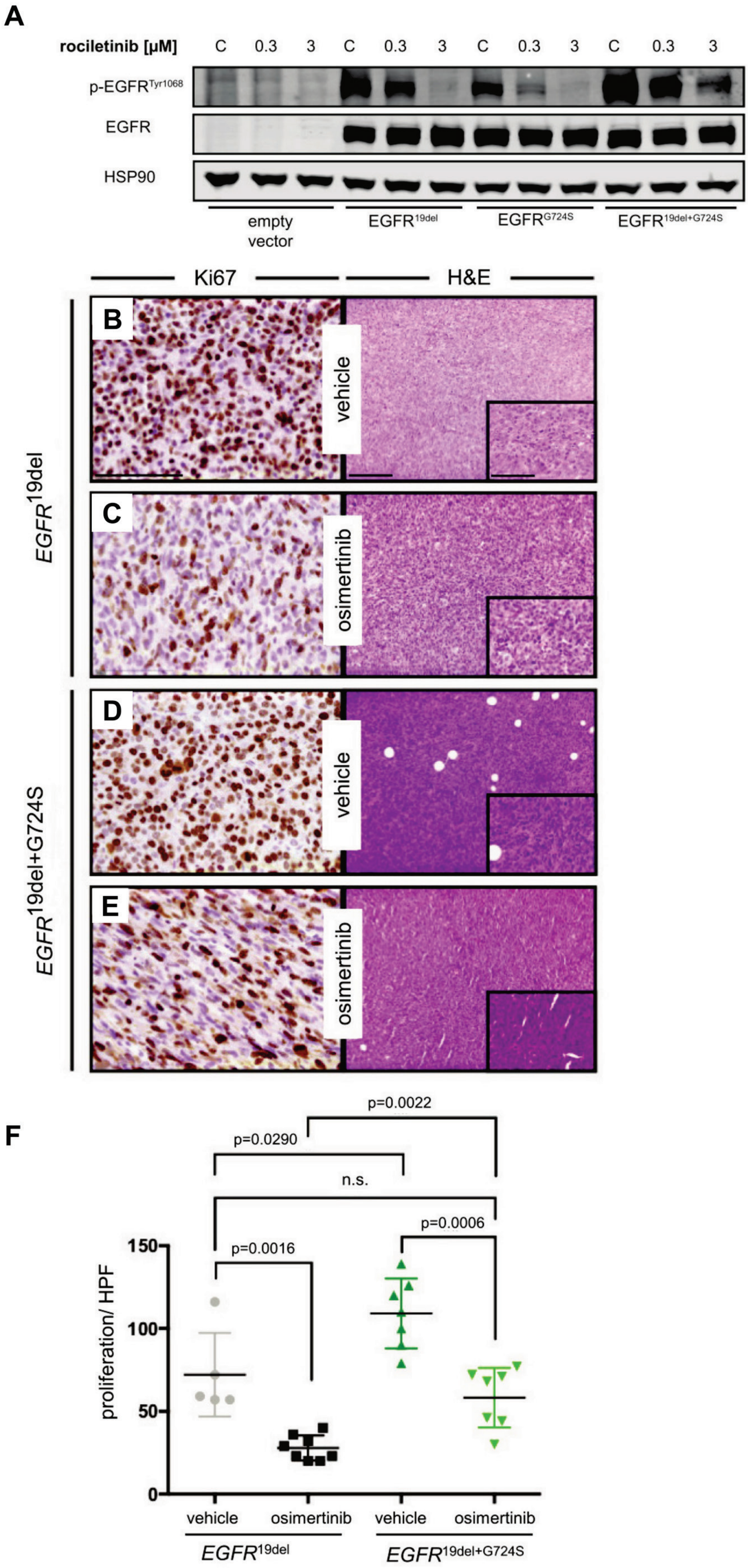
A
rociletinib [µM]
C 0.3 3 C 0.3 3 C 0.3 3 C 0.3 3
p-EGFR Tyr1068
EGFR
HSP90
empty vector
EGFR 19del
EGFR G724S
EGFR 19del+G724S
Ki67
H&E
B
C
D
E
vehicle
osimertinib
vehicle
osimertinib
EGFR 19del
EGFR 19del+G724S
F
p=0.0022
p=0.0290
n.s.
p=0.0006
p=0.0016
proliferation/ HPF
150
100
50
0
vehicle
osimertinib
vehicle
osimertinib
EGFR 19del
EGFR 19del+G724S

Supplementary Figure 2. Rociletinib treatment of NIH-3T3 cells and xenograft model with either canonical EGFR- or additional $EGFR^{G724S}$-mutation. **A)** Immunoblotting results of NIH-3T3 cells (empty vector, $\textit{EGFR}^{19del}$, $\textit{EGFR}^{G724S}$ or $\textit{EGFR}^{19del+G724S}$) monitoring phospho-EGFR and total-EGFR under rociletinib treatment (24h). HSP90 was used as a loading control. **B-E)** Proliferation of subcutaneous tumors with either canonical EGFR- or additional $EGFR^{G724S}$-mutation. Representative pictures of either proliferation marker Ki67 or H&E staining. **F)** Quantification of supplement figure 2A-D. High-power-field (HPF, 400 X), n.s.: non-significant (n = 7-9; Mann-Whitney-U-Test; scale bars Ki67 100 µm, scale bars H&E overview 200 µm, scale bars H&E zoom-inlay 50 µm).

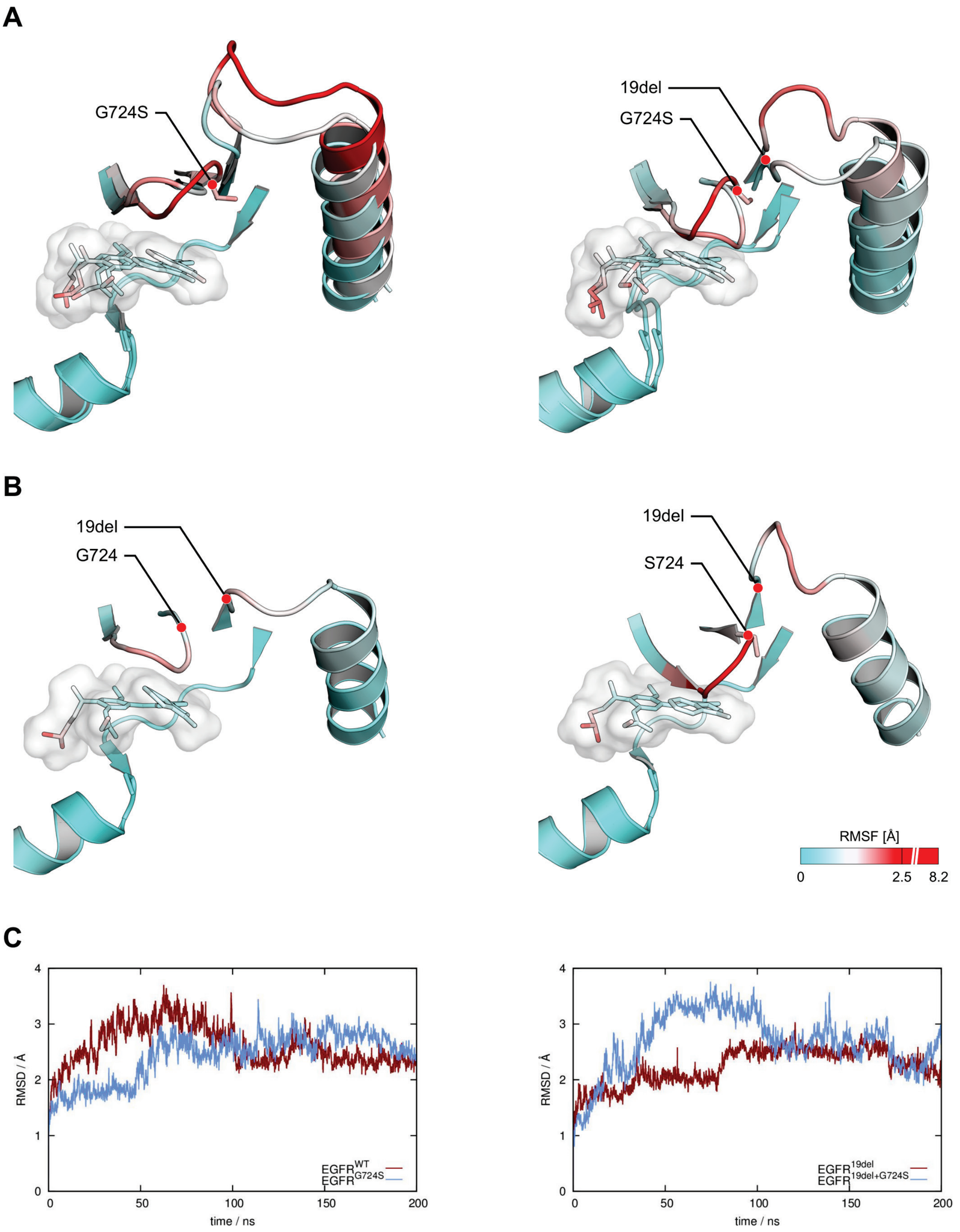

Supplementary Figure 3. Molecular dynamics simulations of EGFR and its mutant variants. **A)** Alignment of molecular dynamics (MD) simulations of osimertinib bound EGFRWT, and EGFRG724S (left) and of osimertinib bound EGFR19del, and EGFR19del+G724S (right) (based on PDB ID: 4ZAU). **B)** Comparison of alternate MD simulations of osimertinib bound EGFR19del (left)

and EGFR19del+G724S (right) corresponding to Figure 3D (left) and 3D (right), started at the respective final frames of EGFRWT and EGFRG724S simulations. Structures were generated similar as before (DBSCAN cutoffs of 1.43 Å). **C)** Root mean square deviations (RMSD) of Cα atoms of the osimertinib–EGFRWT and –EFGRG724S complexes (left) and of the osimertinib–EGFR19del and –EFGR19del+G724S complexes (right) over production simulation time.

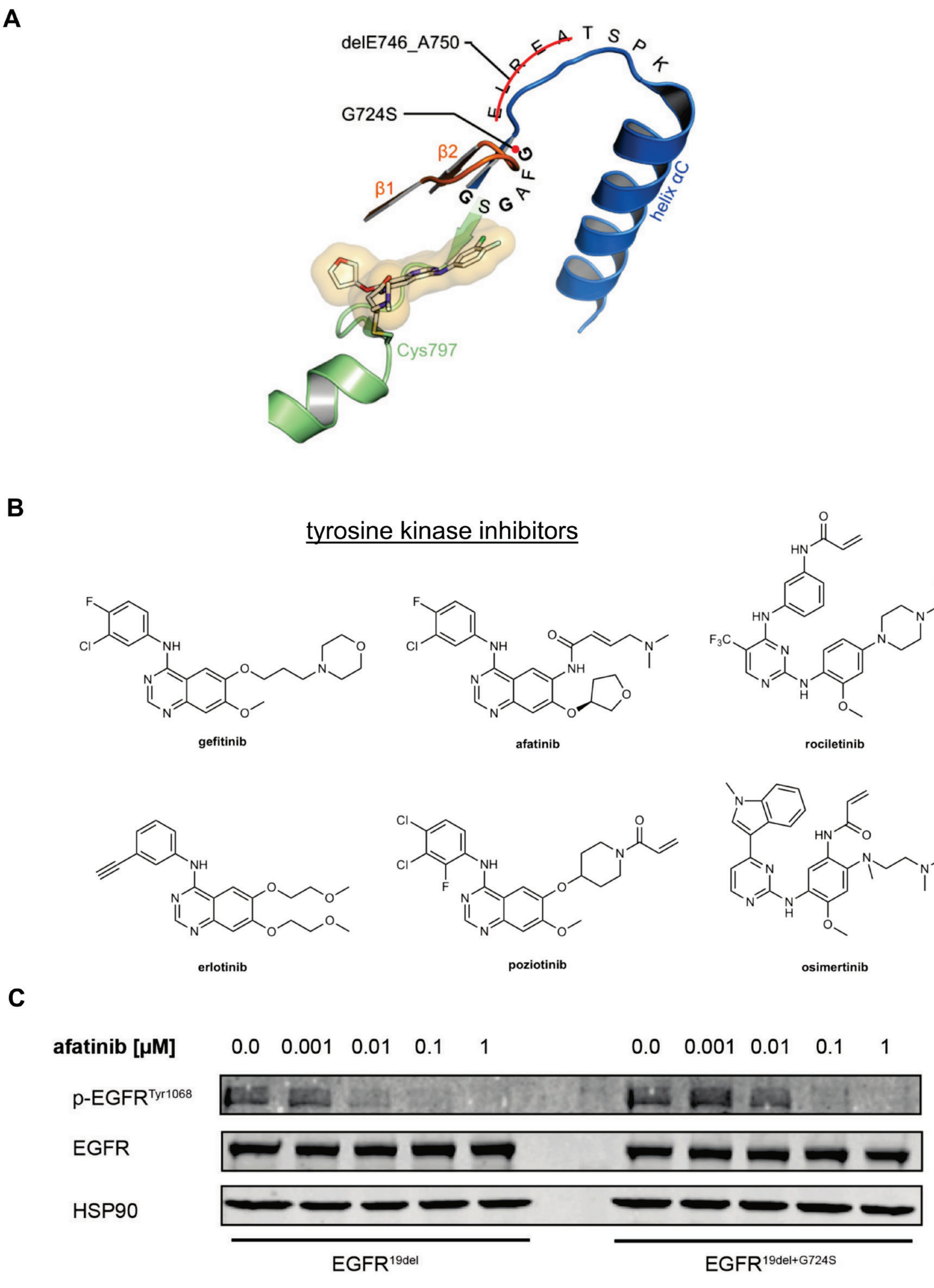

Supplementary Figure 4. Biochemical profiling of EGFRG724S. **A)** Binding site of afatinib bound EGFR (PDB ID: 4G5P). The hinge region is painted in green, the glycine-rich loop that harbors the EGFRG724S mutation, with the sheets β1 and β2 is highlighted in orange and the regulatory helix αC with the adjacent ELREA-motif is colored in blue. **B)** Chemical structures of representative first-,

second- and third-generation EGFR inhibitors. **C)** Immunoblotting of NIH-3T3 cells treated with different concentrations of afatinib for 4 hours.

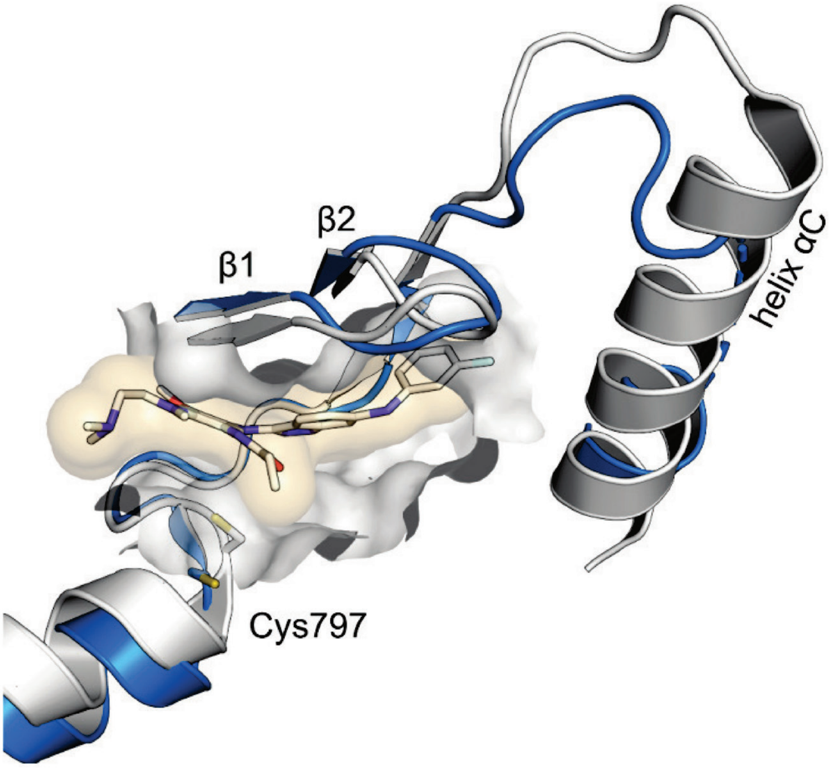

Supplementary Figure 5. Alignment of the aminoindazole-based inhibitor bound cSrc binding site (model based on PDB ID: 5D11) with exon 20 mutant EGFR (white, PDB ID: 4LRM).

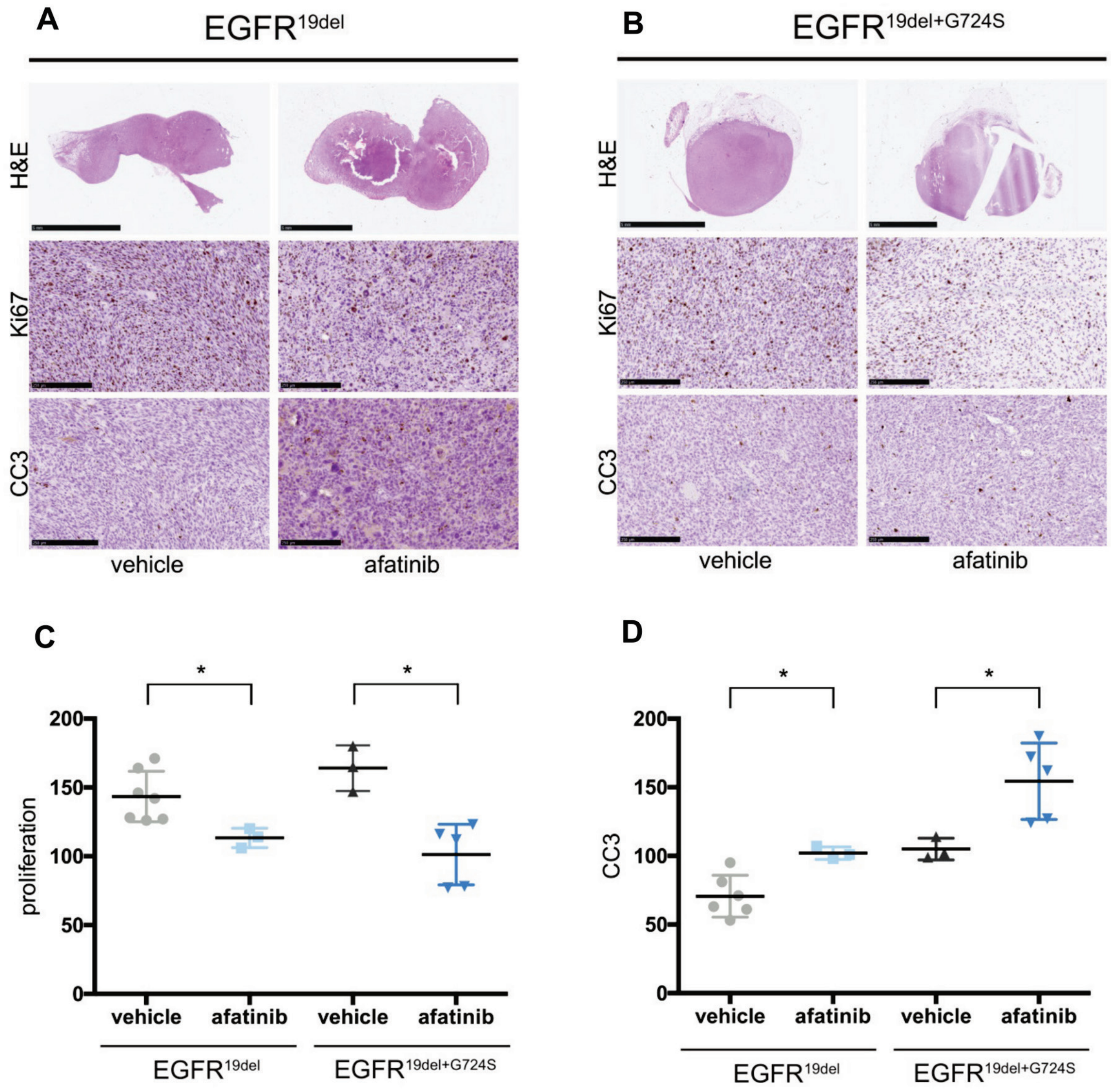

Supplementary Figure 6. Xenograft models treated with afatinib **A)** Representative pictures of either H&E, Ki67 or CC3 staining of tumors of mice bearing NIH-3T3 EGFR19del. **B)** Representative pictures of either H&E, Ki67 or CC3 staining of tumors of mice bearing NIH-3T3 EGFR19del+G724S. **C)** Quantification of proliferation. **D)** Quantification of Cleaved-Caspase-3 staining. * = $p < 0,05$.

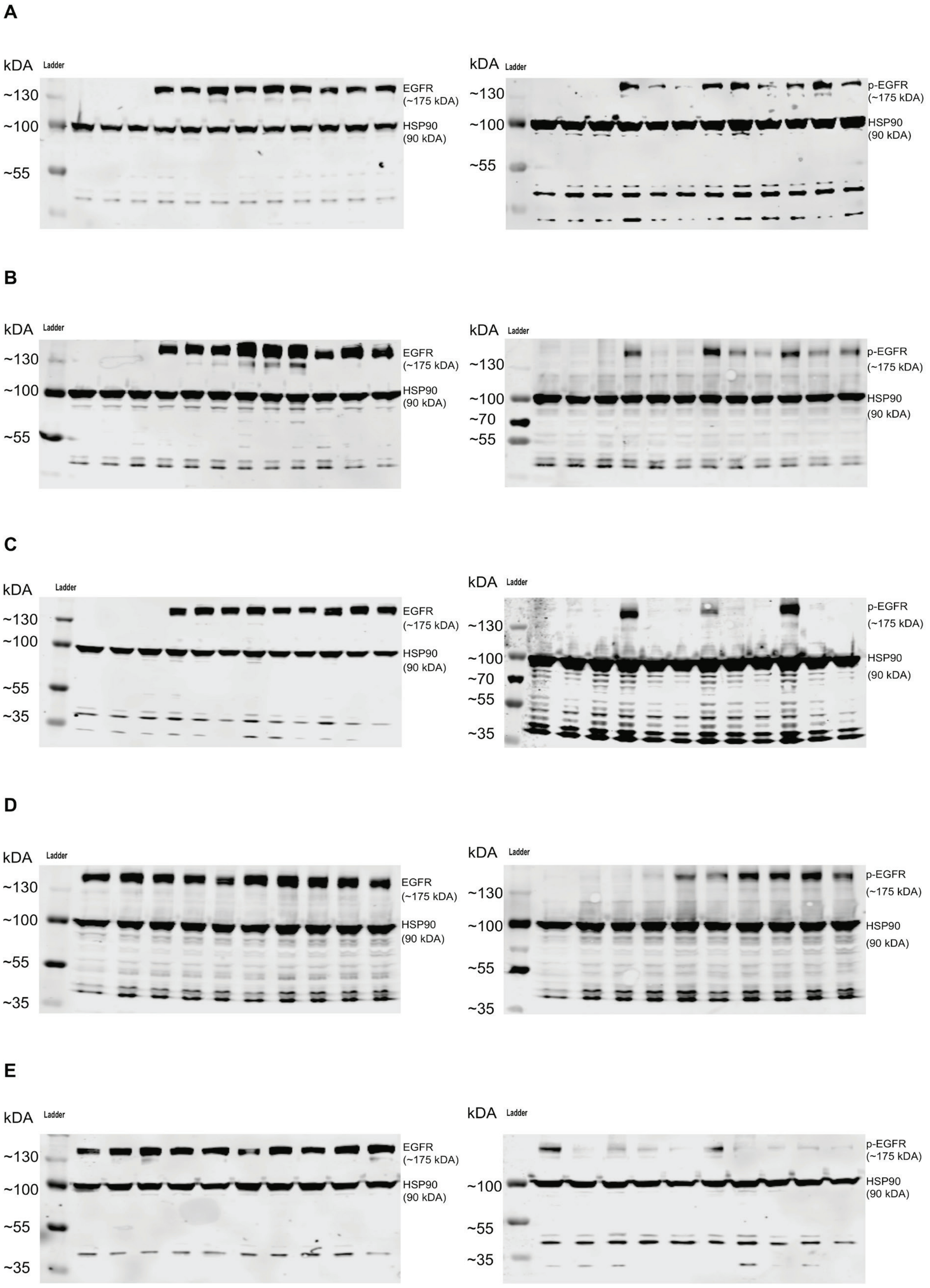

Supplementary Figure 7. Uncropped immunoblotting results. NIH-3T3 cells expressing either empty vector, EGFR19del, EGFRG724S or EGFR19del+G724S treated with erlotinib (A), osimertinib (B) or

afatinib (C) for 24 hours showing total-EGFR (left) and phospho-EGFR (right). D) NIH-3T3 cells expressing EGFR19del or EGFR19del+G724S treated with osimertinib (D) or afatinib (E) for 0, 1, 3, 6 or 24 hours showing total-EGFR (left) and phospho-EGFR (right).

Supplementary Table 1: Summary of the results of the molecular analyses and radiologic assessments by patient.

P1 (female, ns, aad: 59, UICC stage III, OS: 77, 7 month)													
Time point	Prior treatment	Progression as per RECIST	Best response§ as per RECIST	Progressive lesion	Lesion biopsied	NGS performed	Primary EGFR mutation (MF)	EGFR p.T790M (MF)	EGFR p.G724S (MF)	MET ampl.	HER2 ampl.	Other aberrations	Transition
T0	n.a.	n.a.	n.a.	n.a.	unk.	yes	p.E746_S752delinsV (42%)	no	no	n.d.	no	no	no
T1	erlotinib	n.d.	n.d. (PR)	lung, left lower lobe	lung, left lower lobe	yes	p.E746_S752delinsV (Sanger)	yes (Sanger)	no	no	n.d.	no	no
T2	osimertinib	yes	PR	pleural effusion	pleural effusion	yes	p.E746_S752delinsV (8,6%)	no	yes (6,3%)	n.d.	n.d.	no	no

P2 (female, ns, aad: 47, UICC stage IV, OS: 51, 4 month)													
Time point	Prior treatment	Progression as per RECIST	Best response§ as per RECIST	Progressive lesion	Lesion biopsied	NGS performed	Primary EGFR mutation (MF)	EGFR p.T790M (MF)	EGFR p.G724S (MF)	MET ampl.	HER2 ampl.	Other aberrations	Transition
T0	n.a.	n.a.	n.a.	n.a.	unk.	no	p.S752_I759del (Sanger)	n.d.	n.d.	n.d.	n.d.	n.d.	no
T1	erlotinib	n.d.	n.d. (PR)	lung, right lower lobe	lung, right lower lobe	yes	p.S752_I759del (88,3%)	yes (89,8%)	no	no	no	no	no
T2	EGF816	yes	SD	liver	liver	yes	p.S752_I759del (EPI: 82.2%; EP II: 71,2%)	yes (EPI: 39.3%)/no (EPII)	no (EPI)/yes (EPII: 71,1%	no (both)	no (both)	no (both)	no
T3	osimertinib	n.d.	n.d. (PR)	pleural effusion	pleural effusion	yes	p.S752_I759del (48,5%)	no	yes (59,1%)	unevaluable	unevaluable	no	no

P3 (female, ns, aad: 68, UICC stage I, OS: 47, 4 month)													
Time point	Prior treatment	Progression as per RECIST	Best response§ as per RECIST	Progressive lesion	Lesion biopsied	NGS performed	Primary EGFR mutation (MF)	EGFR p.T790M (MF)	EGFR p.G724S (MF)	MET ampl.	HER2 ampl.	Other aberrations	Transition
T0	n.a.	n.a.	n.a.	n.a.	lung, right lower lobe	yes	p.E746_T751delinsIP (48,4%)	no	no	no	no	no	no
T1	gerfitinib	n.d.	n.d. (PR)	lung, right lower lobe	lung, right lower lobe	yes	p.E746_T751delinsIP (42,5%)	yes (6,9%)	yes (5,3%)	no	no	no	no
T2	osimertinib	yes	PR	lung, right lower lobe	lung, right lower lobe	yes	p.E746_T751delinsIP (48,6%)	no	yes (49,6%)	no	no	TP53 p.180*	no

P4 (male, fs, aad: 60, UICC stage IV, OS: 31, 4 month)													
Time point	Prior treatment	Progression as per RECIST	Best response§ as per RECIST	Progressive lesion	Lesion biopsied	NGS performed	Primary EGFR mutation (MF)	EGFR p.T790M (MF)	EGFR p.G724S (MF)	MET ampl.	HER2 ampl.	Other aberrations	Transition
T0	n.a.	n.a.	n.a.	n.a.	unk.	yes	p.E746_S752delinsV (21%)	no	no	no	no	no	no
T1	erlotinib	n.d.	n.d. (PR)	lung, left lower lobe	lung, left lower lobe	yes	p.E746_S752delinsV (39,2%)	yes (6,7%)	yes (14,1%)	low-level	no	TP53 p.R248W	no
T2	osimertinib	n.d.	n.d. (PD)	lung, left lower lobe	lung, left lower lobe	yes	p.E746_S752delinsV (55,5%)	no	yes (38,7%)	no	no	TP53 p.R248W	no

*adj. cisplatin/vinorelbine; **Carboplatin/pemetrexed/bevacizumab + maintenance; PD not determined by RECIST; §In brackets () according to investigator's assessment, RECIST analysis not feasible due to inadequate baseline assessment; MF: molecular fraction; ampl.: amplification; n.a.: not applicable; n.d.: not done; NGS: next generation sequencing; OP: overall survival; PFS: progression-free survival; PR: partial response; SD: stable disease; ToT: time on treatment; UICC: Union International Contre le Cancer.

Supplementary Table 2. Corresponding IC_{50} values for first-, second- and third-generation EGFR inhibitors on *EGFR*$^{19del+G724S}$ shown in Figure 5B.

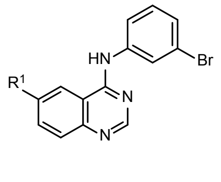

1

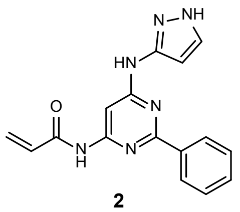

2

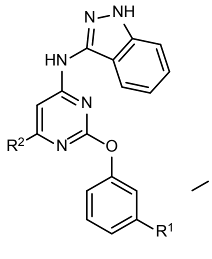

3

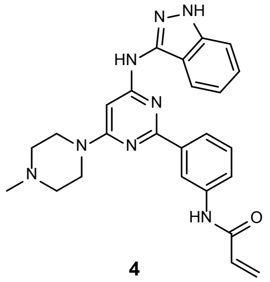

4

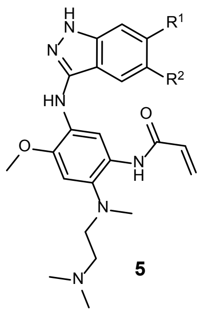

5

TKI ge-neration	compound	R^1	R^2	*EGFR*$^{19del+G724S}$ IC_{50} [nM]
**1**	gefitinib			40.3 ± 7.1
**1**	erlotinib			49.4 ± 9.3
**1**	vandetanib			25.8 ± 8.6
**1**	lapatinib			91.4 ± 17.5
**1**	**1a**	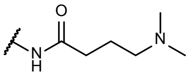		49.8 ± 4.1
**1**	**1b**	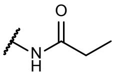		50.8 ±8.7
**2**	afatinib			2.1 ± 1.0
**2**	pelitinib			<1
**2**	allitinib			<1
**2**	poziotinib			<1
**2**	canertinib			<1
**2**	dacomitinib			<1
**3**	osimertinib			33.7 ± 3.7
**3**	rociletinib			41.0 ± 9.5
3	olmutinib			55.3 ± 3.4
**3**	WZ4002			31.1 ± 7.5
**3**	EGF816			68.4 ± 10.1
**3**	naquotinib			>2.000
**3**	brigatinib			112.0 ± 25.5
3	ibrutinib			91.4 ± 27.9
**3**	**2**			1282.5 ± 420.8
**3**	**3a**	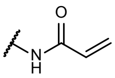	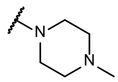	540.4 ± 69.9
**3**	**3b**	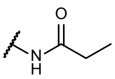	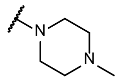	>2.000
**3**	**3c**	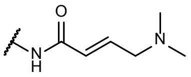	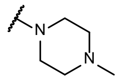	>2.000
**3**	**3d**	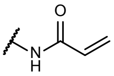	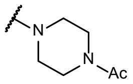	459.3 ± 67.3

**3**	**4**			>2.000
**3**	**5a**	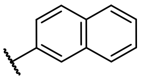	H	>2.000
**3**	**5b**	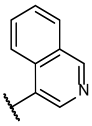	H	844.5 ± 98.4
**3**	**5c**	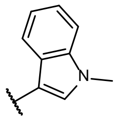	H	255.6 ± 30.4
**3**	**5d**	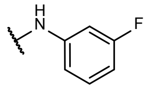	H	3.0 ± 1.2
**3**	**5e**	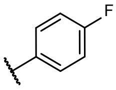	H	50.8 ± 2.2
**3**	**5f**	H	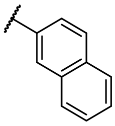	106.5 ± 8.0

Values are the mean ± SD of three independent measurements in duplicates.

Supplementary Table 3. Overview of IC_{50} values and kinetic parameters K_i, k_{inact} and k_{inact}/K_i determined for first-, second- and third-generation EGFR inhibitors on $EGFR^{WT}$, $EGFR^{L858R}$, $EGFR^{L858R+T790M}$, $EGFR^{19del}$, $EGFR^{19del+G724S}$.

compound	EGFR	IC_{50} [nM]	K_i [nM]	k_{inact} [min^{-1}]	k_{inact}/K_i [µM^{-1}s^{-1}]
	WT	<1			
	L858R	<1			
**gefitinib**	L858R+T790M	190.4 ± 100.3			
	19del	2.4 ± 0.6			
	19del+G724S	40.3 ± 7.1			
	WT	<1			
	L858R	<1			
**erlotinib**	L858R+T790M	163.6 ± 8.3			
	19del	4.8 ± 1.6			
	19del+G724S	49.4 ± 9.3			
	WT	<1	<1	0.07 ± 0.01	>1.2*
	L858R	<1	<1	0.04 ± 0.01	>1.2*
**afatinib**	L858R+T790M	1.3 ± 0.1	1.9 ± 0.1	0.13 ± 0.01	1.07 ± 0.05
	19del	<1	<1	0.05 ± 0.02	>1.2*
	19del+G724S	2.1 ± 1.0	<1	0.03 ± 0.01	>1.2*
	WT	<1	<1	0.05 ± 0.02	>1.2*
	L858R	<1	<1	0.01 ± 0.02	>1.2*
**poziotinib**	L858R+T790M	<1	1.1 ± 0.1	0.30 ± 0.03	4.42 ± 0.65
	19del	<1	<1	0.07 ± 0.03	>1.2*
	19del+G724S	<1	<1	0.02 ± 0.01	>1.2*
**osimertinib**	WT	2.3 ± 0.2	14 ± 2.3	0.43 ± 0.11	0.52 ± 0.05

	L858R	<1	1.6 ± 0.3	0.30 ± 0.01	3.24 ± 0.46
	L858R+T790M	<1	1.5 ± 0.1	0.33 ± 0.06	3.75 ± 0.39
	19del	<1	<1	0.14 ± 0.03	>1.2*
	19del+G724S	33.7 ± 3.7	80.4 ± 35.7	0.19 ± 0.08	0.04 ±0.00
	WT	2.2 ± 0.5	74 ± 7.1	0.18 ± 0.01	0.04 ± 0.01
	L858R	2.0 ± 0.2	1.8 ± 0.2	0.18 ± 0.05	1.67 ± 0.32
**rociletinib**	L858R+T790M	3.3 ± 0.6	1.7 ± 0.1	0.29 ± 0.05	2.95 ± 0.66
	19del	2.3 ± 0.3	4.5 ± 0.1	0.12 ± 0.01	0.48 ± 0.05
	19del+G724S	41.0 ± 9.5	164.3 ± 37.3	0.25 ±0.07	0.03 ± 0.00

Values are the mean ± SD of three independent measurements in duplicates. *quotient was determined with K_i = 1 nM.
