# Supplementary material for: Overcoming EGFRG724S-mediated osimertinib resistance through unique binding characteristics of second-generation EGFR inhibitors
Source: Nat Commun. 2018 Nov 7;9:4655. doi: 10.1038/s41467-018-07078-0 (PMC6220297; doi:10.1038/s41467-018-07078-0)
Supplement: Supplementary file 2 — Description of Additional Supplementary Files [file 41467_2018_7078_MOESM2_ESM.pdf]

Description of Additional Supplementary Files

File Name: Supplementary Data 1
Description: (EGFR_WT_osimertinib_calcBfac298.pdb): MDgenerated structure of osimertinib bound to EGFR_WT

File Name: Supplementary Data 2
Description: (EGFR_G724S_osimertinib_calcBfac298.pdb): MDgenerated structure of osimertinib bound to EGFR_G724S

File Name: Supplementary Data 3
Description: (EGFR_19del_osimertinib_calcBfac298.pdb): MDgenerated structure of osimertinib bound to EGFR_19del

File Name: Supplementary Data 4
Description: (EGFR_19del_G724S_osimertinib_calcBfac298.pdb):
MD-generated structure of osimertinib bound to EGFR_19del_G724S
